# Estrogen-induced chromatin decondensation and nuclear re-organization linked to regional epigenetic regulation in breast cancer

**DOI:** 10.1186/s13059-015-0719-9

**Published:** 2015-08-03

**Authors:** Sehrish Rafique, Jeremy S. Thomas, Duncan Sproul, Wendy A. Bickmore

**Affiliations:** MRC Human Genetics Unit, Institute of Genetics and Molecular Medicine, University of Edinburgh, Crewe Road South, Edinburgh, EH4 2XU UK; Edinburgh Breakthrough Research Unit and Edinburgh Cancer Centre, University of Edinburgh, Western General Hospital, Crewe Road South, Edinburgh, Scotland EH4 2XU UK

## Abstract

**Background:**

Epigenetic changes are being increasingly recognized as a prominent feature of cancer. This occurs not only at individual genes, but also over larger chromosomal domains. To investigate this, we set out to identify large chromosomal domains of epigenetic dysregulation in breast cancers.

**Results:**

We identify large regions of coordinate down-regulation of gene expression, and other regions of coordinate activation, in breast cancers and show that these regions are linked to tumor subtype. In particular we show that a group of coordinately regulated regions are expressed in luminal, estrogen-receptor positive breast tumors and cell lines. For one of these regions of coordinate gene activation, we show that regional epigenetic regulation is accompanied by visible unfolding of large-scale chromatin structure and a repositioning of the region within the nucleus. In MCF7 cells, we show that this depends on the presence of estrogen.

**Conclusions:**

Our data suggest that the liganded estrogen receptor is linked to long-range changes in higher-order chromatin organization and epigenetic dysregulation in cancer. This may suggest that as well as drugs targeting histone modifications, it will be valuable to investigate the inhibition of protein complexes involved in chromatin folding in cancer cells.

**Electronic supplementary material:**

The online version of this article (doi:10.1186/s13059-015-0719-9) contains supplementary material, which is available to authorized users.

## Background

While genetic aberrations altering gene expression and genomic stability are a hallmark of cancer, epigenetic changes are also frequently observed and have the potential to be crucial influences on carcinogenesis [1]. Epigenetic alterations have been mostly explored at the single gene level but there are increasing reports of contiguous genes being coordinately repressed in association with tumor progression — a phenomena known as long-range epigenetic silencing (LRES) [2, 3]. Both focal and regional epigenetic alterations are likely to contribute to the heterogeneity of cancer.

The tendency of genes that are clustered in the genome to be co-expressed has long been noted in many eukaryotic genomes [4] and has been suggested to be influenced by the chromatin and nuclear environments across a chromosomal domain [5]. Indeed, coordinate gene regulation has been linked to lamin-associated domains (LADs), regional chromatin compaction [6] and to topologically associated domains (TADs) [7]. However, for the most part the mechanisms underlying the coordination of expression from clustered genes remain unclear.

Coordinately dysregulated clusters of genes have been reported in association with chromosomal abnormalities [8]; however, the best described and understood instances of long-range gene dysregulation come from cancer. In these instances, LRES has been most commonly identified by detecting DNA methylation at the promoters of clustered genes [9–14]. Some of these studies have been extended to show that decreased gene expression in these regions is accompanied by the loss of histone modifications associated with gene activity (e.g., H3K4me3) [9, 15] and the gain of repressive histone marks — H3K9 methylation, H3K27me3 and histone hypoacetylation [10, 15, 16]. Gene repression associated with these epigenetic alterations does not necessarily involve the acquisition of DNA methylation [17].

More recently, in prostate cancer long-range epigenetic activation (LREA) of genes has been reported, associated with a loss of H3K27me3 and a gain of H3K9ac [18]. The mechanism of activation is not clear but it was suggested that it might involve DNA methylation of promoter-associated CpG islands and transcription from alternative promoters.

In bladder carcinoma, expression data were used to uncover LRES regions by determining the correlation of each gene’s expression profile with that of its neighbors [19]. Comparative genome hybridization (CGH) data were used to exclude regions where coordinately reduced expression was due to copy number aberrations. LRES has been identified in a wide-range of epithelial cancers (bladder, colorectal, prostate, gastric). Furthermore, the LRES phenotype can be specific to subsets of bladder cancer and correlates with tumor stage and aggressiveness [17]. In some breast tumors, epigenetic silencing of HOXA and protocadherin gene clusters was reported [9, 11]. There was no explicit investigation of tumor subtype, although the two breast cancer cell lines investigated (MDAMB231 and Bt 549) happen to be of the basal-B subtype [20].

By integrating analysis of coordinate gene expression, DNA methylation and data on estrogen receptor alpha (ERα) binding sites in the MCF7 breast cancer cell line, 11 regions of LRES were reported in association with estrogen signaling [21]. For one region (16p11.2), coordinate repression was estrogen-inducible in normal breast epithelial cells and was associated with the formation of 3C (chromosome conformation capture) associations that were interpreted as a large looped chromatin structure bringing together the promoters of the 14 silenced genes [21].

To determine whether higher-order chromatin organization is more generally linked to the coordinate dysregulation of genomic regions in breast cancer and whether this is associated with tumor subtype, we have identified regions of regional epigenetic regulation (RER) that are independent of copy number alterations in breast tumors and breast cancer cell lines. As well as regions of LRES, we found regions of LREA in tumors relative to normal breast tissue. We demonstrate that regional gene expression differences within one LREA region — present in both breast tumors and cell lines — are associated with alterations in chromatin compaction and nuclear organization. Chromatin at this region is visibly less compact in ER-positive (ER+) breast cancer cells that have the RER phenotype, compared with the ER-negative (ER−) tumor subtypes and to normal breast epithelium. In MCF7 cells, we show that at this locus, estrogen is responsible for inducing chromatin decompaction and a more central position in the nucleus. This study highlights the importance of studying regulation beyond the level of single genes and suggests that as well as alterations to DNA methylation and histone modifications, aberrant chromatin organization may contribute to genome dysregulation in cancer.

## Results

### Regions of copy number-independent transcriptional correlation in breast tumors

**Fig. 1:**
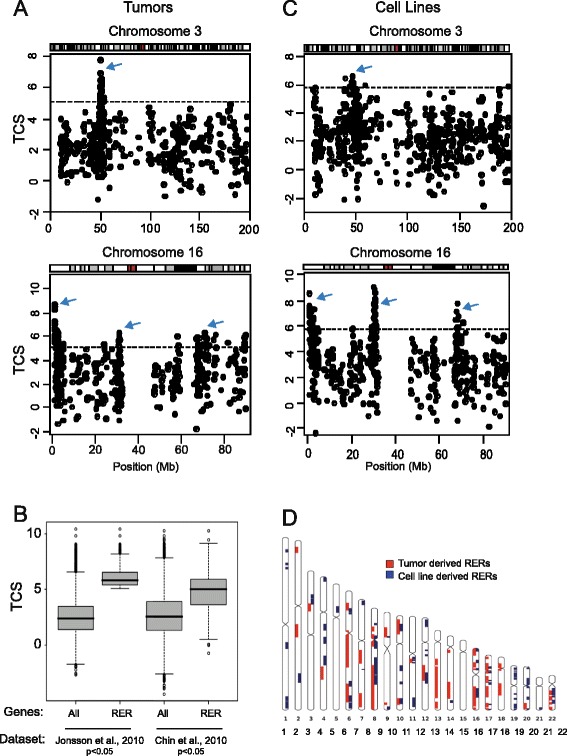
Identifying RER in breast tumors and cancer cell lines. **a**, **c** Transcription correlation score (TCS) maps for chromosomes 3 and 16 using data from breast tumors [22] (**a**) and breast cancer cell lines [20] (**c**). The *horizontal dotted line* indicates the significance threshold. *Arrows* indicate regions containing genes with significant TCSs. **b** Boxplots showing the distribution of TCSs generated for all genes and for RER genes using two independent breast tumor datasets [22, 23]. **d** Ideograms showing the location of the 45 RER regions identified in breast tumors (*red*) and the 71 RER regions identified in breast cancer cell lines (*blue*)

To identify chromosomal regions containing genes that are coordinately expressed independent of genomic changes in breast cancer, we implemented an approach based on that used to find LRES regions in bladder cancer [19]. We applied this to the analysis of transcription (expression microarray) and copy number (array CGH) from 356 breast tumors [22]. To ensure apparent RER regions were not caused by copy number variation, we excluded from further analysis genes for which a copy number aberration was detected in that sample. A transcription correlation score (TCS) was calculated for each gene to quantify how well its expression correlated with that of its neighbors. This score was the sum of the Spearman rank correlations for a given gene’s expression with that of each of its ten nearest neighbors. TCS maps generated from this tumor set revealed peaks corresponding to potential RER regions (Fig. 1a, arrows; Figure S1 in Additional file 1).

We identified 382 genes with significantly outlying high TCSs (*p* < 0.05, threshold TCS 5.08, false discovery rate (FDR) 6.6 % by permutation). The expression patterns of genes with significant TCSs were significantly correlated with that of their individual neighboring genes (mean 15.66, range 10-20, Spearmans rho, *p* < 0.05). Furthermore, they also possessed high TCSs in a second independent set of breast tumors [23] (Fig. 1b), demonstrating that our result was not particular to the dataset analyzed. We merged adjacent significant TCS windows to delineate 45 RER regions that included at least two genes with significant scores. These regions ranged in size from 0.12 Mb to 43 Mb (median 1.86 Mb) (Table S1 in Additional file 1).

Consistent with a coordinately regulated gene expression signature, the identified RER regions included one (6p22.1-p22.3) that contains the histone gene cluster whose expression is coordinately regulated in early S phase [24]. This region also includes *GMNN*, which encodes the replication licensing inhibitor geminin and whose expression also peaks in S phase [25].

Overall, RER in breast cancer occurs in regions of the genome that are significantly more gene dense than expected by chance (Figure S2a in Additional file 1). Analysis of Gene Ontology associated with the significant TCS genes highlighted terms associated with metabolic processes and with the regulation of the EGFR/ERRB pathway, known to be very important in the biology of breast cancer (Figure S2b in Additional file 1). Five subunits of the mediator complex, which is involved in transcriptional regulation, especially by nuclear receptors [26], are encoded by genes with significant TCSs in four RER regions. Seven genes encoding mitochondrial ribosomal proteins have significant TCSs in six RER regions (Table S1 in Additional file 1). Expression of genes involved in mitochondrial biogenesis and function, and especially those encoding mitochondrial ribosomal proteins, is particularly elevated in epithelial cancer cells [27].

### RER regions show differential expression in breast tumor subtypes

**Fig. 2:**
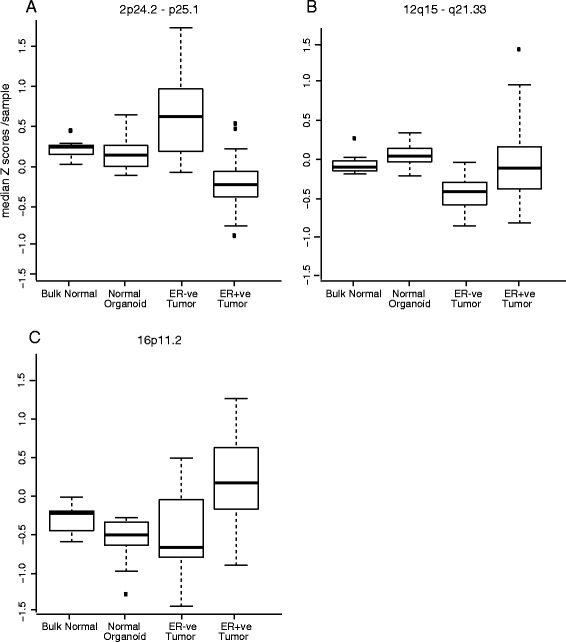
Gene expression changes in tumors and normal tissue. Box plots show the distribution of mean centered z scores of gene expression in tumor samples and normal breast tissue and breast organoids [28] for genes in the RER regions at 2p24.2-p25.1 (**a**), 12q15-q21.33 (**b**) and 16p11.2 (**c**). Data for tumors are separated according to ER status and Wilcoxon tests were used to determine whether or not there was a significant difference between tumor and normal samples taken together

We compared gene expression levels in the RER regions in breast tumors with those in bulk normal breast tissues, using datasets from [28, 29] that also include expression data derived from breast organoid preparations which are enriched in the epithelial cells known to give rise to tumors. There were examples where RER region expression was significantly (*p* < 0.05) up-regulated in ER− and down-regulated in ER+ tumors (2p24.2-p25.1; Fig. 2a), or vice versa (18q12.3-q21.32), relative to normal tissue or organoids. An RER region at 12q15-q21.33 (Fig. 2b) was down-regulated relative to normal only in ER− tumors and one at 14q23.3-q32.11 had a similar pattern only in ER+ tumors. Elevated expression in ER− tumors only (i.e., no significant change in ER+ tumors) relative to normal organoid was seen in two instances (16q12.2-q24.1 and 20q13.2-q13.33). Expression was up-regulated in ER+ tumors only, relative to normal, in a total of 12 RER regions, (e.g., RER region 16p11.2; Fig. 2c). Finally, expression was up-regulated in both tumor types relative to normal in 13 RER regions.

**Fig. 3:**
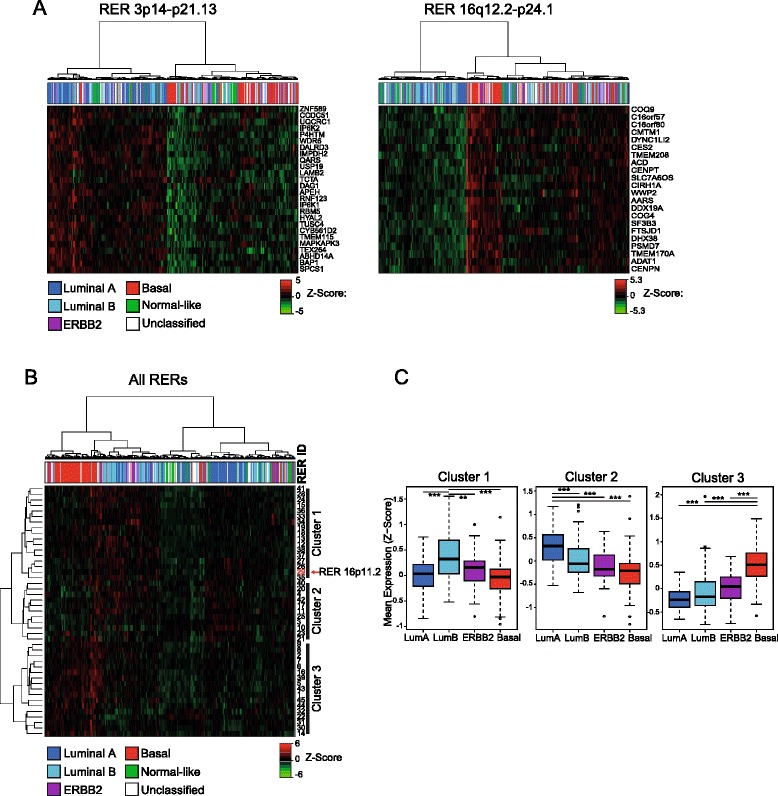
Properties of RER regions and tumor subtypes. **a** Unsupervised hierarchical cluster analysis of breast tumor samples for RER regions at 3p14-p21.31 (*left*) and 16q12.2-q24.1 (*right*). Heat maps of gene expression z scores with hierarchical clustering of samples (*red* high expression, *green* low expression). Genes are ordered by their position in the genome. Subtype information [22] for each tumor sample is identified by the color-coded matrix: luminal A (*blue*), luminal B (*turquoise*), ERBB2 (*purple*), basal (*red*), normal-like (*green*). Only genes in the regions with significant TCSs are shown. **b** As in (**a**) but for mean expression (mean z score of genes with significant TCSs) for all RER regions in each breast tumor sample, showing clustering of RER regions into three groups. Both the RER regions and samples were subject to hierarchical clustering. **c** Box plots showing mean expression (mean z score of genes with significant TCSs) of RER regions from clusters 1, 2 and 3 in breast tumors of different subtypes; luminal A (*LumA*, *blue*), luminal B (*LumB*, *turquoise*), ERBB2 (*purple*), basal-like (*red*). **p < 0.01, ***p < 0.001, Wilcoxon test)

To better understand the patterns of coordinate gene expression in relation to tumor biology, we examined heat maps of gene expression data for significant TCS genes in RER domains. For many of these regions, unsupervised hierarchical clustering separated breast tumors by the intrinsic subtypes previously defined by gene expression [30] (e.g., luminal and basal-like) and revealed cases where there is a tumor subtype-specific gene signature (activation or repression) within RER regions. For example, an RER region at 3p14-p21.31 (Fig. 3a) has elevated expression in luminal (ER+) relative to basal tumors (ER−), whereas one at 16q12.2-q24.1 (Fig. 3b) is repressed in luminal ER+ relative to basal type tumors.

To determine whether the RER regions we detected in breast tumors are independent of each other or whether they might be co-expressed in the same tumor, we analyzed the mean expression patterns of the 45 RER regions and found that they fall into three co-expressed clusters (Fig. 3b). The highest mean expression of cluster 1 RER regions is detected in luminal B subtype tumors whereas cluster 2 RER regions are preferentially expressed in luminal A tumors, and cluster 3 RER regions in basal-like tumors (Fig. 3c).

### RER domains in breast cancer cell lines

To gain more mechanistic insight into the factors affecting the formation of RER regions in a tractable experimental system, we also generated TCS maps for 48 breast cancer cell lines [20]. This revealed 557 genes grouped into 71 regions of copy number-independent transcriptional correlation, 0.1–15.8 Mb (median 0.9. Mb) in size (Fig. 1c; Figure S3 and Table S2 in Additional file 1). The smaller average RER region size in cell lines compared with tumors likely reflects the better genome coverage of the expression array platforms used for the former. Apart from this difference, transcription correlation maps from breast tumors and cell lines were quite similar (Fig. 1c).

In total we identified 26 copy number-independent regions of coordinate expression (0.23–13.4 Mb in size (median 1.40 Mb)) that are in common between breast tumors and breast cancer cell lines (Table 1). Gene expression was up-regulated relative to normal breast in nine of these RER regions and it was down-regulated in a further eight regions. The remaining regions showed no significant change in expression between cancer and normal cells, i.e., at these genomic regions coordinate gene regulation is either typical of both the normal and the cancerous state or is balanced out overall by changes in different directions in different tumor subtypes (e.g., Fig. 2a).

**Table 1:** Cytogenetic band(s) where RER regions common to breast tumors and breast cancer cell lines are located

Cytogenetic location	Size (Mb)	Number of significant TCSs (tumor/cell line)	Number of other significantly correlated genes (tumor/cell line)	Genes in RER regions with significant TCSs	Gene expression (tumor versus normal)
6p22.2	0.89	3/34	14/28	*C6orf62*, *GMNN*, *HIST1H4C*/Histone gene cluster, *ZSCAN16*, *GPX5*, *OR2W1*, *OR2J3*, *OR2N1P*, *OR12D2*, *OR10C1*, *MOG*	Up
6q23.2-q23.3	3.43	12/3	26/10	*TAAR5*, *TAAR3*, *VNN3*, *IFNGR1*, *HEBP2*, *ABRACL*, *HECA*, *VTA1*, *PEX3*, *FUCA2*, *LTV1*, *SHPRH*, *PPIL4*, *RMND1*, *C6orf211*	Down
6q25.1-q25.3	6.71	12/3	26/7	*RP23-468K3.1*, *RP3-527B10.1*, *OPRM1*	Down
8p11.21-q11.23	13.41	6/6	17/8	*TM2D2*, *GOLGA7*, *KAT6A*, *AP3M2*, *IKBKB*, *POLB*, *VDAC3*, *SLC20A2*, *SMIM19*, *MCM4*, *MRPL15*	Down
8q21.13-q21.3	10.54	32/6	33/11	*NBN*, *OTUD6B*, *RAD54B*, *KIAA1429*, *ESRP1*, *INTS8*, *PLEKHF2*, *MTERFD1*, *PTDSS1*, *MTDH*, *HRSP12*, *AP003355.2*, *VPS13B*, *ANKRD46*, *UBR5*, *AZIN1*, *ATP6V1C1*, *SLC25A32*, *TTC35*, *TAF2*, *MRPL13*, *DERL1*, *ATAD2*, *WDYHV1*, *TRMT12*, *RNF139/KCNB2*, *STAU2*, *FAM164A*, *STMN2*, *FAM82B*, *MMP16*	No change
8q22.1-q23.1	11.99	32/11	33/3	*NBN*, *OTUD6B*, *RAD54B*, *KIAA1429*, *ESRP1*, *INTS8*, *PLEKHF2*, *MTERFD1*, *PTDSS1*, *MTDH*, *RPL30*, *HRSP12*, *AP003355.2*, *NIPAL2*, *VPS13B*, *COX6C*, *SPAG1*, *RNF19A*, *ANKRD46*, *UBR5*, *AZIN1*, *ATP6V1C1*, *FZD6*, *SLC25A32*, *EMC2*, *TAF2*, *MRPL13*, *DERL1*, *ATAD2*, *WDYHV1*, *TRMT12*, *RNF139*	No change
9p13.3	1.13	2/6	20/11	*NOL6*, *SIGMAR1*, *KIAA1045*, *DNAJB5*, *RUSC2*, *CD72*, *SIT1*, *CA9*	No change
10p13-p12.31	6.71	5/3	22/10	*UPF2*, *CDC123*, *HSPA14*, *RPP38*, *NMT2*, *RSU1*, *STAM*	Down
10q26.11-q26.13	6.34	3/5	21/10	*BAG3*, *C10orf119*, *SEC23IP*, *BRWD2*, *PLEKHA1*, *IKZF5*	No change
11q12.3-q13.1	0.75	5/2	27/9	*C11orf48*, *WDR74*, *COX8A*, *OTUB1*, *MACROD1*, *NUDT22*, *RPS6KA4*	Up
11q13.2	0.36	6/2	18/9	*KAT5*, *FIBP*, *CCDC85B*, *SART1*, *SF3B2*, *YIF1A* , *SPTBN2*, *C11orf80*	Up
12q21.31-q21.33	10.39	2/4	17/12	*RAB21*, *PPP1R12A* , *NTS*, *MGAT4C*, *DCN*, *EEA1*	Down
16p13.3	2.16	23/23	33/22	*AXIN1*, *TMEM8A*, *NME4*, *RAB11FIP3*, *PIGQ*, *RAB40C*, *LA16c-398G5.2*, *WDR90*, *RHOT2*, *WDR24*, *METRN*, *FAM173A*, *CCDC78*, *NARFL*, *IFT140*, *NME3*, *MRPS34*, *HAGH*, *NDUFB10*, *GFER*, *NTHL1*, *TRAF7*, *MLST8*, *E4F1*, *GNG13*, *LMF1*, *CACNA1H*, *TPSG1*, *TPSD1*, *UBE2I*, *BAIAP3*, *PGP*	Up
16p13.3	1.45	4/5	16/12	*OR1F1*, *OR2C1*, *NAA60*, *ADCY9*, *TFAP4*, *DNAJA3*, *ANKS3*, *ROGDI*, *UBN1*	Up
16p12.3	0.38	3/2	13/6	*KNOP1*, *GP2*, *EARS2*, *NDUFAB1*, *PALB2*	No change
16p11.2	1.36	6/25	22/13	*SEZ6L2*, *TAOK2*, *HIRIP3*, *DOC2A*, *ALDOA*, *PPP4C*, *TBX6*, *MAPK3*, *CD2BP2*, *TBC1D10B*, *ZNF48*, *ZNF771*, *ZNF768*, *ZNF747*, *ZNF764*, *ZNF688*, *ZNF785*, *PRR14*, *FBRS*, *SRCAP*, *PHKG2*, *RNF40*, *ZNF629*, *BCL7C*, *CTF1*, *SETD1A*, *VKORC1*	Up
16q22.1-q22.2	4.9	22/11	71/17	*CDH16*, *NOL3*, *E2F4*, *ATP6V0D1*, *THAP11*, *PSKH1*, *DDX28*, *DUS2L*, *PRMT7*, *COG4*, *VAC14*	Down
17p11.2	1.69	17/3	26/6	*TOM1L2*, *LRRC48*, *LLGL1*	Down
17q11.2	0.53	17/4	26/6	*UNC119*, *KIAA0100*, *SDF2*, *SUPT6H*	Up
17q21.2	0.28	17/6	13/9	*KRTAP1-3*, *KRTAP1-1*, *KRTAP2-4*, *KRTAP4-9*, *KRT34*, *KRT31*	No change
17q25.1	0.9	24/4	20/6	*KCTD2*, *GGA3*, *MRPS7*, *GRB2*	Up
17q25.3	0.58	13/4	18/13	*STRA13*, *RFNG*, *CSNK1D*, *SECTM1*	Up
22q11.23-q12.1	5.93	5/13	15/20	*TRMT2A*, *P2RX6*, *TOP3B*, *PPIL2*, *IGLV1-40*, *ZNF280B*, *ZNF280A*, *ZDHHC8P*, *VPREB3*, *MMP11*, *UPB1*, *SEZ6L*, *CRYBB1*	Down
22q12.2-q12.3	1.01	3/2	15/11	*INPP5J*, *PIK3IP1*	No change
22q12.3	0.24	3/4	15/12	*TMPRSS6*, *SSTR3*, *MFNG*, *GCAT*	No change
22q13.1-q13.2	1.37	2/6	16/10	*CBX6*, *APOBEC3A*, *PDGFB*, *MGAT3*, *CACNA1I*, *SGSM3*	No change

The RER region size (measured as the first to last significant TCS in the region), the number of genes with significant TCSs in the tumor or cell line data, the number of other genes in the region which have significantly correlated expression with the TCS genes (again for tumors and cell lines), and the list of those genes with significant TCSs. The gene expression of the region in tumors compared with normal samples is also indicated

**Fig. 4:**
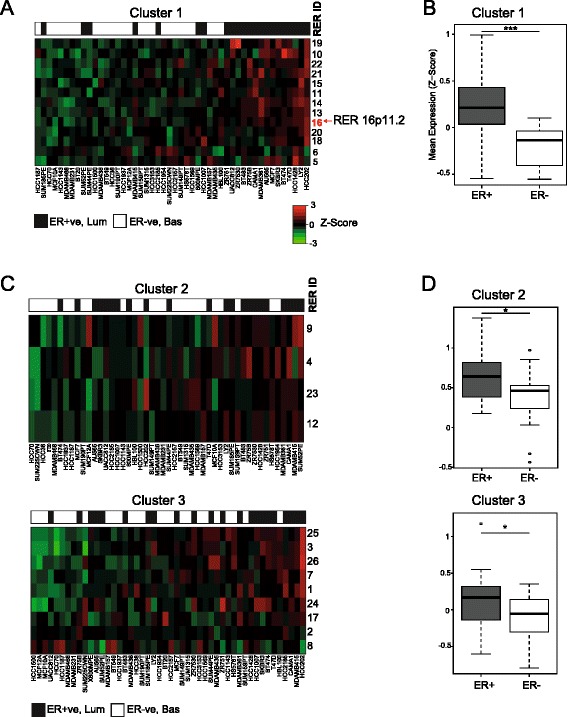
Properties of RER regions in breast cancer cell lines. Analysis of mean expression (mean z score of genes with significant TCSs) levels in breast cancer cell lines for cluster 1 RER regions (**a**) and RER regions of clusters 2 and 3 (**c**). RER regions were subject to hierarchical clustering and cell lines were ordered by their overall level of expression of each RER cluster. Box plots showing mean expression (mean z score of genes with significant TCSs) of RER regions from cluster 1 (**b**) and clusters 2 and 3 (**d**) in ER+ (*gray*) and ER− (*white*) breast cancer cell lines (*p < 0.05, ***p < 0.001)

For cell line RER regions equivalent to those from tumor RER regions of cluster 1, mean expression levels were higher in ER+ than in ER− cell lines (Fig. 4a, b). The expression of cluster 2 and 3 RER regions was not so well modeled in the cell lines (Fig. 4c, d). This might reflect the fact that the majority of breast cancer cell lines were established from advanced cancers and thus luminal cell lines would be expected to be equivalent to luminal B tumors (which express cluster 1 RER regions) rather than less aggressive luminal A tumors (which express cluster 2 RER regions). Similarly, many ER− breast cancer cell lines are known to reflect the claudin-low, mesenchymal subtype of breast tumor, which is very rare in vivo [20].

### Chromatin and nuclear reorganization of RER domains

One of the cluster 1 RER regions common to both the tumor and cell line datasets is on chromosome 16p11.2 and encompasses a region previously reported as regulated by LRES in estrogen-responsive breast cancer cells [21]. Our analysis of expression for all genes in this RER region (not just those with a significant TCS) revealed a differential expression pattern between luminal, ER+ and basal, ER− breast cancer subtypes, with increased gene expression in luminal tumors (Fig. 2c). This is replicated in breast cancer cell lines — mean expression levels within this RER region are higher in ER+ breast cancer cell lines than in ER− ones (Fig. 4a, b).

**Fig. 5:**
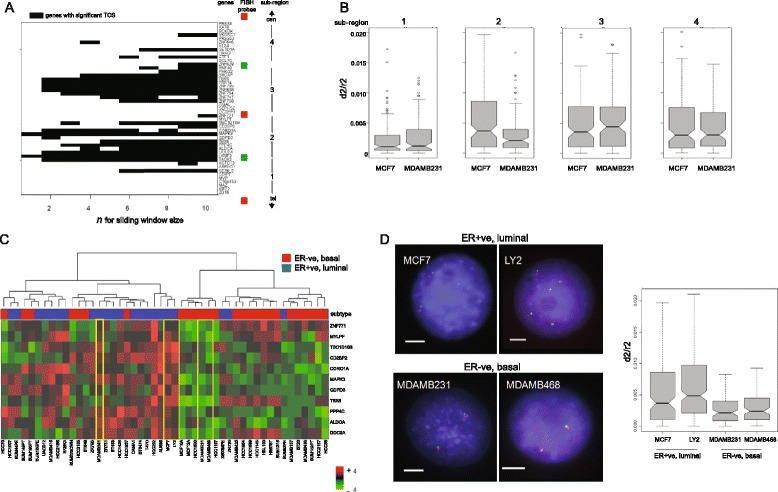
Refinement and analysis of the 16p11.2 RER region in breast cancer cell lines. **a** *Black bars* indicate genes along 16p11.2, oriented from centromere (*top*) to telomere (*bottom*), which have significant TCSs at varying window (2*n* + 1) size with *n* from 1 to 10. Gene expression data are from tumor cell lines [20]. Gene names are listed to the right, as are the position of fluorescence in situ hybridization (FISH) probes that were used to examine the four RER subregions. **b** Box plots show the distribution of normalized FISH interprobe distances (*d* ^*2*^ */r* ^*2*^) [31, 32] measured across the four subregions of the 16p11.2 RER region in MCF7 and MDAMB231 breast cancer cell lines. *n* = 45–60 nuclei. The significance of differences between datasets was assessed by Wilcox test (Table S3 in Additional file 1). **c** Unsupervised cluster analysis of gene expression z scores for subregion 2 in 48 breast cancer cell lines (*red* ER−, *blue* ER+) [20]. Cell line names are indicated along the bottom of the heat map. *Red*/*green* z scores equate to increased/decreased gene expression, respectively. Genes are ordered by their position on the chromosome and listed to the right. The *yellow boxes* indicate cell lines examined by FISH. **d** Example FISH images using probe pairs (*red* and *green*) that delineate subregion 2 (as in (**a**)) in ER+ cell lines MCF7 and LY2 (*upper panels*), and ER− cell lines MDAMB231 and MDAMB468 (*lower panels*). DNA is stained with DAPI (*blue*). Scale bar = 5 μm. The boxplots to the right show the distribution of normalized FISH interprobe distances (*d* ^*2*^ */r* ^*2*^) across subregion 2 in the four cell lines. *n* = 45–60 nuclei. The significance of differences between datasets was assessed by Wilcox test (Table S3 in Additional file 1)

To determine whether 16p11.2 is one contiguous block of RER, or several different subregions, we analyzed TCSs generated by varying the number of neighboring genes (*n*) used in the sliding window analysis (from 10 — the value used for the original analysis – down to 1). As *n* decreased to 8 and below, genes with remaining high TCSs were resolved into distinct two RER subregions (2 and 3) that are located more proximal on 16p11.2 than the LRES region defined by Hsu et al. [21] (subregion 1 in Fig. 5a).

We have previously shown that fluorescence in situ hybridization (FISH) can detect long-range chromatin decompaction that occurs as a result of differentiation, perturbation of epigenetic mechanisms or signaling pathways, or genetic disorders [31–34]. To determine whether the changes in gene expression seen in the 16p11.2 RER region also correspond to altered large-scale chromatin compaction, we performed FISH using probes located at the boundaries of the two ~400 kb subregions defined in Fig. 5a on nuclei of the luminal ER+ MCF7 and basal-type ER− MDAMB231 breast cancer cell lines [20] (subregions 2 and 3). These were compared to two adjacent subregions that were less enriched in genes with significant TCSs (subregions 1 and 4). Analysis of the normalized inter-probe distance (d^2^/r^2^) [32] revealed that only subregion 2 showed a significant (*p* = 0.03, Wilcoxon rank-sum test) difference in chromatin compaction between MCF7 and MDAMB231, with the region being de-compact in MCF7 cells (Fig. 5b; Table S3 in Additional file 1).

Unsupervised hierarchical clustering of expression data from 48 breast cancer cell lines for genes in subregion 2 [20] segregated luminal ER+ and basal ER− cell line subtypes (Fig. 5c). The LY2 derivative of MCF7, which though ER+ has estrogen-independent growth [35], shows elevated gene expression and chromatin decompaction within subregion 2, like parental MCF7s (Fig. 5d; Table S3 in Additional file 1). Conversely, a second ER− breast cancer cell line, MDAMB468, showed reduced gene expression and a compact chromatin structure, like MDAMB231. A less compact chromatin structure in MCF7 and LY2 cells was not seen at negative control loci that are not within an RER region (Figure S4 in Additional file 1).

**Fig. 6:**
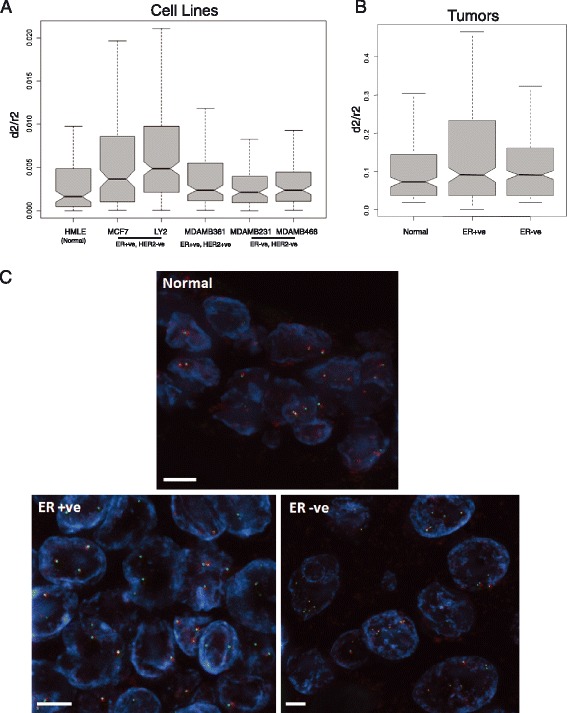
Chromatin compaction at subregion 2 of the 16p11.2 RER region in breast cancer cell lines, in normal breast tissue and in primary breast tumors. **a** Box plots comparing the distribution of normalized FISH interprobe distances (*d* ^*2*^ */r* ^*2*^) measured across subregion 2 of the 16p11.2 RER region in a normal breast cell line (HMLE) and in ER+ (MCF7, LY2, MDAMB361) and ER− (MDAMB231 and MDAMB468) breast cancer cell lines. *n* = 45–60 cells. The significance of differences between datasets was assessed by Wilcox test (Table S3 in Additional file 1). **b** Box plots showing the distribution of normalized FISH interprobe distances (*d* ^*2*^ */r* ^*2*^) measured across subregion 2 of the 16p11.2 RER region in normal breast tissue and in ER+ and ER− tumor tissues. *n* = 250–300 alleles. Distances in the ER+ tumor were significantly greater than in normal tissue (*p* < 0.0001) or in the ER− tumor (*p* = 0.004). Differences between normal and ER− tumor tissue were not significant (*p* = 0.24). **c** Example FISH images using probe pairs (*red* and *green*) that delineate subregion 2 in normal breast tissue and in ER+ and ER− tumor tissue. DNA is stained with DAPI (*blue*). Scale bar = 5 μm

To determine the chromatin compaction status at subregion 2 in a normal breast cell line, FISH was also carried out on the non-transformed immortalized human mammary epithelial cell line HMLE [36]. The chromatin state of this region in HMLE was more compact than in MCF7 and LY2 cells, but not significantly different to that in the ER− cell lines MDAMB231 and MDAMB468 (Fig. 6a). A second independent ER+ breast cancer cell line, MDAMB361, showed a trend to being more de-compact than HMLE but this difference was not significant (Fig. 6a). This lesser de-compaction correlates with the expression level of genes in subregion 2 in MDAMB361, which was lower than in MCF7s and LY2 (Fig. 5c). We also note that unlike MCF7s and LY2 cells, MDAMB361 cells are HER2+ due to copy number amplification of the *ERBB2* oncogene [20]. Our analysis of RER gene expression demonstrates that the breast tumors of the ERBB2 subtype have lower expression levels of cluster 1 RER regions (Fig. 3b). This suggests that the expression of ERBB2 oncogene lessens RER region expression and chromatin decompaction phenotype of cluster 1 RER regions, such as the 16p11.2 region.

To examine chromatin structure at subregion 2 in vivo, 3D FISH was also performed on tissue sections from an ER+ breast tumor, from an ER− tumor and from normal breast tissue. The chromatin at this region of 16p11.2 was most compact in normal tissue, though this was not significantly different from that in the ER− tumor. Chromatin at this region was, however, significantly less compact in the ER+ tumor compared with either the ER− tumor or normal tissue (Fig. 6b, c), confirming that chromatin de-compaction of subregion 2 in ER+ breast cancer is not an artifact of cell culture.

### Estrogen mediates chromatin de-compaction and nuclear re-organization

**Fig. 7:**
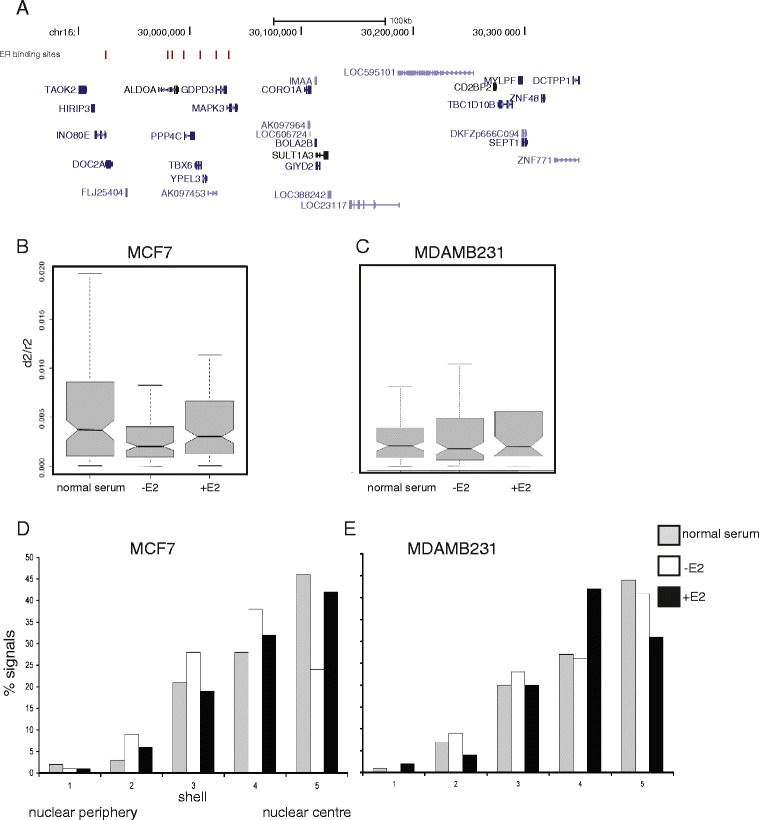
The effect of estrogen on chromatin compaction and nuclear organization at subregion 2 of the 16p11.2 RER region. **a** Map of the 16p11.2 RER subregion 2 showing the location of ER binding sites (*red*) in MCF7 cells (from [38]). Below, the location of genes in the region is shown from the UCSC Genome Browser NCBI36/hg18 assembly of the human genome. **b**, **c** Box plots comparing the distribution of normalized FISH interprobe distances (*d* ^*2*^ */r* ^*2*^) measured across subregion 2 of the 16p11.2 RER region in ER+ MCF7 (**b**) and ER− MDAMB231 (**c**) breast cancer cell lines. Data are shown for cells grown in normal serum, in media stripped of hormone for 3 days (*−E2*), and after addition of 100 nm estrogen for 24 hours (*+E2*). *n* = 60 cells for each sample. **d**, **e** The percentage of FISH hybridization signals for subregion 2 of the 16p11.2 RER region found in each of five shells of equal area eroded from the edge of the nucleus (shell 1) through to the nuclear center (shell 5), in MCF7 (**d**) and MDAMB231 cells (**e**) grown in normal serum (*grey shaded bars*), hormone stripped media (*−E2*, *white*) and after addition of 100 nm estrogen for 24 hours (*+E2*, *black*)

The association between ER status and RER within subregion 2 suggested that estrogen might be responsible for the observed differences in chromatin compaction. As well as inducing local changes in chromatin modification, ER has been reported to be capable of inducing large-scale visible chromatin de-condensation on an artificial reporter array [37]. Examination of chromatin immunoprecipitation (ChIP)-sequencing data from MCF7 cells [38] revealed seven ER-bound sites within the 400 kb subregion 2 of the 16p11.2 RER region (Fig. 7a). A permutation analysis of 10,000 randomly placed genomic windows of equal size to subregion 2 (~414 kb; using BEDtools v.2.17.0) showed that subregion 2 is among the top 6.2 % of regions in the human genome in terms of enrichment for ER binding sites.

To test whether the de-compact chromatin state of subregion 2 in MCF7 cells depends on estrogen, MCF7 and the ER− breast tumor cell line MDAMB231 were cultured in phenol-free media with fetal calf serum (FCS) that had been stripped of all endogenous hormones (−E2 in Fig. 7). This resulted in a significant (*p* = 0.002) compaction of chromatin at subregion 2 in MCF7 cells (Fig. 7b), but not in MDAMB231 (*p* = 0.41) (Fig. 7c). The cells were then treated with 100 nM 17β-estradiol (estrogen, E2) for 24 hours (+E2 in Fig. 7). These conditions activate high-level expression of estrogen-regulated genes in MCF7 cells [39]. E2 treatment resulted in chromatin de-compaction of subregion 2 in MCF7 cells relative to the –E2 conditions (*p* = 0.003) and a return to a chromatin compaction state similar to that seen in MCF7 cells grown in normal serum (*p* = 0.52). Addition of E2 to MDAMB231 cells had no effect on chromatin compaction in this region (*p* = 0.32). Chromatin de-compaction in MCF7 cells upon the addition of E2 was not seen at a control locus outside of the 16p11.2 locus 2 RER region (Figure S5 in Additional file 1).

As well as changes in chromatin condensation, the radial position of some genes in the nucleus has been linked to their activity [33, 40]. We therefore quantified the radial nuclear position of subregion 2 of the 16p11.2 RER region across five shells of equal area eroded from the periphery (shell 1) through to the center (shell 5) of the nucleus in MCF7 cells. As expected, given the known preferred position of gene-rich human chromosome 16 toward the center of the nucleus [41], hybridization signals from subregion 2 were predominantly found in the nuclear center (Fig. 7d). Hormone deprivation led to a significant re-localization of the region away from the nuclear center, and central nuclear localization was restored by the re-addition of estrogen. In contrast, in the ER− cell line MDAMB231 the removal of hormone by growth in stripped media did not affect the localization of the 16p11.2 RER region and the re-addition of estrogen resulted in the locus adopting a less central position in the nucleus (Fig. 7e).

These data are consistent with the hypothesis that the de-compact higher-order chromatin state and the maintenance of a central nuclear localization of subregion 2 in ER+ breast cancer cells with an RER phenotype is mediated by the action of estrogen itself.

## Discussion

### Regional epigenetic regulation in breast cancer

Dysregulation of gene expression is a common event in cancer, and a number of long-range events have been documented in various solid tumors. These studies have generally uncovered large chromosomal domains associated with gene repression and are accompanied by a cocktail of cancer-associated epigenetic changes in DNA methylation and histone modifications associated with repression [2, 3, 9–17]. Less frequently documented has been the coordinate up-regulation of genes in chromosomal domains in cancer [18].

Here we identify regional epigenetic regulation that is present in breast tumors and breast cancer cell lines. We found regions of copy-number independent coordinate down-regulation of gene expression (LRES) relative to expression levels reported in normal breast tissue, and also regions of coordinate up-regulation (LREA). Twenty-six RER regions were found to be in common between tumors and cancer cell lines. In addition, the RER regions we identify fall into three groups characterized by being expressed primarily in different breast cancer subtypes.

Genes in pathways previously implicated in tumor biology are present in RER regions, so understanding the mechanisms that lead to RER formation is important. In bladder cancers, a multiple regional epigenetic silencing phenotype was found to occur in a subset of aggressive tumors of the carcinoma in situ pathway but not in tumors driven by mutations in *FGFR3* [17]. Here, we also found that RER regions often segregate with tumor subtype, with some RER regions being associated with breast tumors of the luminal ER+ subtype, and others found in the basal ER− subtype. None of the RER regions we identified as in common between breast tumors and breast cancer cell lines overlap with those identified in bladder carcinoma [19]. However, five of the RER regions identified in breast tumors, but not in breast cancer cell lines, overlap those identified in bladder cancer (Table S4 in Additional file 1). This includes the domain at 3p22.3 that was found to be associated with increased histone methylation (H3K9me3 and H3K27me3), histone hypoacetylation and a compact chromatin structure in bladder cancer [16, 17].

Twelve RER regions identified here in breast tumors overlap regions of LRES found in prostate cancer [3] and two of these (at 8q22.3-q23.1 and 10q26.13) are also in common with RER regions found in breast cancer cell lines. One of the regions of LREA reported in prostate cancer [18] overlaps the RER region at 12q21.31-q21.33 identified here. However, in breast cancers (ER−) this region seems to be down-regulated (i.e., subject to LRES) compared with normal breast tissue (Fig. 2b).

These comparisons suggest that there are regions of the human genome prone to recurrent RER in the context of different epithelial cancers. This might be due to the underlying mechanisms that give rise to RER being particularly prone to dysregulation, and/or it could reflect selection for dysregulation of genes in these regions during tumorigenesis.

### RER regions do not seem to correspond to TADs

**Fig. 8:**
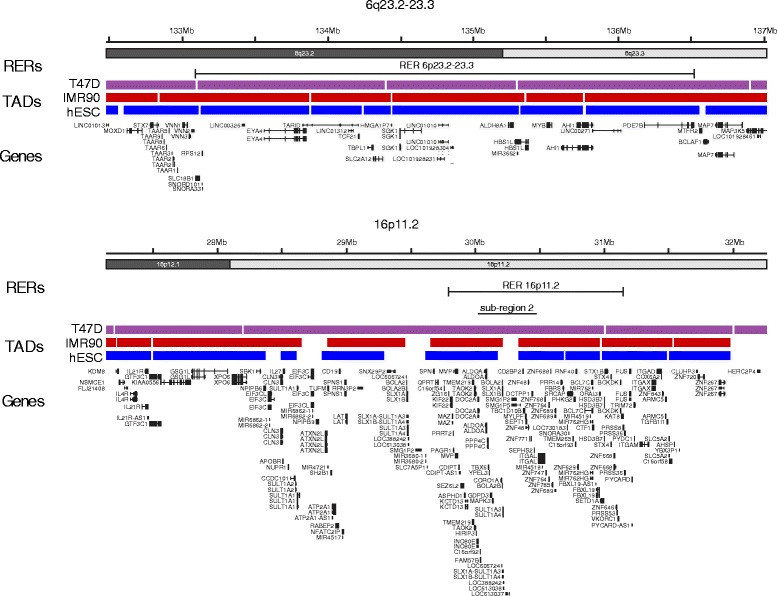
RER regions and TADs do not overlap. Diagrams of the RER regions at 6q23 (*top*) and 16p11.2 (*bottom*), showing the extent of the two RER regions and the location of TADs in the T47D breast cancer line (*purple*), IMR90 fibroblasts (*red*) and human ESCs (*blue*). TAD data are from [43, 44]

The median size of the RER regions that we identified in breast cancer cells lines is similar (900 kb) to the average size of TADs that have been defined in mammalian genomes from the ligation frequencies in Hi-C and 5C experiments [42]. Indeed, it has been suggested that TAD structure allows for coordinate gene regulation [7]. Hi-C analysis is not available for the breast cancer cell lines that we have analyzed by FISH here, but overall TAD structure is remarkably similar between very diverse human cell types. Therefore, we analyzed the degree of overlap between the RER regions defined here and TADs identified in human embryonic stem cells (hESCs) and IMR90 fibroblasts [43] as well as in the T47D breast cancer cell line [44]. The latter cell line does not show an RER phenotype at the 16p11.2 locus in our analysis (Fig. 5c), but extensive coordinate gene regulation in response to progesterone in these cells is reported to generally occur within TADs. However, even using a relaxed threshold of 80 % overlap between a RER region and a single TAD, we found that few of our RER regions correspond to single TAD domains; six (23 %) for TADs in hESCs, eight (31 %) for IMR90 and ten (38.5 %) for T47D (Fig. 8a). Bootstrapping with randomly repositioned RER domains shows that this overlap is not significantly different to that expected by chance. Subregion 2 of the 16p11.2 RER region — the main focus of study in this manuscript — spans a TAD boundary in hESCs and IMR90 cells, but is contained within one larger TAD from the T47D breast cancer cell lines (Fig. 8b). We conclude that our breast cancer RER regions do not correspond to TADs. However, we cannot exclude the possibility that this is because our analyses of RER regions and TADs are based on data from different cell lines or potentially because TADs are disrupted in cancer.

### An RER region at 16p11.2

One of the RER regions we identified as common to both breast tumors and breast cancer cell line datasets (16p11.2) encompasses the region previously reported as regulated by LRES in estrogen-responsive breast cancer cells [21] (Fig. 5). That study identified 11 domains of LRES that were estrogen-mediated in breast cancer; however, only the one at 16p11.2 is a significant RER domain in our analyses of breast cancer cell lines and tumors. The mechanism suggested to underpin this coordinate gene repression was large-scale DNA looping. However, in our analysis this 530 kb region (subregion 1 in Fig. 5) shows up-regulation of gene expression relative to normal breast tissue (Fig. 2c).

Further examination of the 16p11.2 region, using smaller window sizes for the transcription correlation analysis (*n* < 10 genes), showed that the region of epigenetic dysregulation could be resolved into two separate blocks, which we designated as subregions 2 and 3 and which are located more centromere proximal than subregion 1 (Fig. 5a). Subregion 3 contains a cluster of KRAB-zinc finger genes, which are known to form a large chromatin domain coated in the heterochromatin protein CBX1 (HP1β) and in the H3K9 methyltransferase SUV39H1 [45]. Subregion 2 contains a number of genes involved in cell proliferation and signaling (*TAOK2*, *PPP4C*, *MAPK2*) as well as two genes (*HIRIP3* and *INO80E*) involved in chromatin assembly and nucleosome remodeling.

### The ER and large-scale chromatin organization

Using 3C techniques, it has previously been suggested that subregion 1 of the 16p11.2 RER region involves 14 gene promoters in a stable DNA loop structure that is a physically repressive barrier to transcription in cancer cells, including MCF7s [21]. However, using FISH we found no significance difference in chromatin compaction at this region, between ER+ MCF7 and ER− breast cancer cell lines, that might be consistent with such a chromatin structure (Fig. 5b). Cross-linked associations captured by 3C methods have been reported that do not necessarily correspond to spatial proximity as assayed by FISH [46] and might even be indicative of cross-linking not directly between the sequences concerned, but indirectly through association to a common nuclear compartment [47].

In contrast, we did find a significant visible difference in long-range chromatin structure between ER+ (MCF7 and LY2) and ER− (MDAMB231 and MDAM468) breast cancer cell lines that have RER signatures at 16p11.2 (Fig. 5c). This altered chromatin structure was confined to subregion 2 (Fig. 5b, d). Chromatin across this region was less compact in MCF7 and LY2 cells than ER− cell lines and a normal mammary epithelial cell line (Fig. 5a). Moreover, this region was also less compact in an ER+ primary tumor tissue sample than in normal breast tissue or an ER− tumor (Fig. 5c). A second ER+ cell line, MDAMB361, showed a lesser, non-significant trend towards de-compaction. This correlates with overexpression of *ERBB2* due to copy-number amplification and lower expression of subregion 2 genes in MDAMB361, and with the lower expression of cluster 1 RER regions like 16p11.2 in ERBB2 breast tumors. Therefore, it is possible that *ERBB2* expression leads to a lessening of the estrogen-mediated de-compaction in subregion 2. Crosstalk between ERBB2 and estrogen signaling has long been observed in breast cancer and ERBB2 overexpression has been associated with estrogen-independent growth of ER+ breast cancer cell lines and resistance to endocrine therapy in breast tumors [48–50].

Subregion 2 contains a high concentration of binding sites for the ER (Fig. 6a) [38]. As well as altering histone modifications and decondensing local chromatin structure [51], the ER has also been shown to visibly de-compact large-scale chromatin architecture by recruiting coactivators [37]. ER mainly binds at distal elements away from target genes [52]; therefore, its ability to operate over a long range is key to its function. The work reported here suggests that the reach of ER on chromatin structure is further than previously thought, and results in ligand-dependent chromatin unfolding. This is at odds with the suggested formation of compact looped chromatin structures as deduced from cross-linking frequencies obtained by 3C-type methods [21], but is consistent with the observed ability of ER to unfold large-scale chromatin structures at transgene loci [37]. The unfolding of higher-order chromatin at a region of LREA, which we describe here, is redolent of the chromatin compaction at regions of LRES that we recently reported in specific bladder cancer subtypes [16].

ER binding sites are concentrated in the distal 100 kb of subregion 2 of the 16p11.2 RER region, yet transcription correlation spreads over a larger (400 kb) domain. As well as effects on chromatin folding, we also found that estrogen affects the radial position of this chromosomal domain and this is consistent with recent evidence linking chromatin unfolding to radial nuclear organization [53]. Altered radial nuclear localization brought about as a result of genomic rearrangement has been suggested to result in long-range changes of gene expression on the chromosome concerned [54]; thus, we speculate that both altered chromatin folding and nuclear localization may contribute to the long-range epigenetic effects underlying the regional influence on gene expression at the 16p11.2 RER region. We also note that altered nuclear localization of specific genes has been reported in breast cancer [55] and our study extends this finding to larger genomic regions.

## Conclusions

Copy-number independent coordinate dysregulation of gene expression over large chromosome regions is found in breast cancers and is specific to tumor subtype. For one region of up-regulated gene expression in ER+ luminal cancer this is linked to estrogen-dependent unfolding of higher-order chromatin structure (chromatin de-compaction) and a relocalization within the nucleus in MCF7 cells.

## Materials and methods

### Ethics

Use of tumor material was approved by the Lothian Research Ethics Committee (08/S1101/41), obtained under the auspices of Experimental Cancer Medicine Centre program (Edinburgh). Formalin-fixed paraffin-embedded samples were obtained from a tissue bank and were fully anonymized under the same approval.

### Gene expression and CGH data sets

Oligonucleotide arrays (NCBI Gene Expression Omnibus (GEO), platform GPL5345) and bacterial artificial chromosome (BAC) microarrays (GEO platform GPL4723) consisting of 32,000 clones were used for global analysis of gene expression and copy number in 359 breast tumors [22]. All gene identifiers were mapped to Ensembl annotations using Ensembl BioMart. Where multiple probes were mapped to a gene the probe with the highest median expression was used.

By examining copy number profiles for 145 primary breast tumors using Scanning (2464 BACs at 1 Mb intervals) and OncoBAC arrays (960 P1-derived artificial chromosome (PAC), or BAC clones), and gene expression profiles for 130 breast tumors (Affymetrix U133A arrays), data were obtained for an additional dataset of 96 tumors [23].

Expression data for 42 invasive ductal carcinoma samples and 143 breast tissue samples with normal histopathology were obtained from published Affymetrix U133Plus 2.0 GeneChip data [28]. Raw data were processed using standardized Robust Multi-array Average (RMA) normalization (Bioconductor Affy package) and mapped to Ensembl gene annotations.

Expression profiles for 51 breast cancer cell lines were obtained using Affymetrix U133A array and copy number data using Scanning and OncoBAC arrays [20]. Expression and copy number data for 48 common cell lines were used for analysis. Raw data were processed using RMA normalization (Bioconductor Affy package) and mapped to Ensembl gene annotations.

CGH calling was done using the ‘R’ package CGHcall [56]. Genome coordinates for CGH clones were mapped to the UCSC Human Genome Browser (build 37/hg19) and to their nearest ENSEMBL gene in the expression data for the respective tumor or cell line. Copy number-affected genes were then removed from the corresponding expression dataset for that sample to produce a copy number-independent gene expression file that was used for all subsequent analyses.

### Transcription correlation scores

The running score method, used previously to identify regions of LRES in bladder cancer [19], was adopted here. For each gene a TCS was calculated from the sum of the Spearman rank correlation scores between the RNA levels of a gene with that of each of its neighbors. A sliding window approach (2*n* + 1 where *n* = number of neighbors either side of gene) was used to calculate scores for all genes. A Quantile-Quantile (Q-Q plot) indicated a normal data distribution but with a tail of outliers at the high end that would be indicative of genes in windows of coordinate regulation. Significance was assessed using z scores and a significance threshold set with *p* < 0.05 (for z scores unlikely to be observed in a normal distribution characterized by the mean and standard deviation of the TCSs observed).

RER regions were then delineated by extracting *n* number of genes either side of the significant TCS genes (to get all the genes in the sliding window). Regions containing less than two significant TCS genes were discarded and overlapping regions were merged together. Regions were further refined by calculating the median gene expression value for each gene across all samples in the dataset and working out the correlation between this median and the value for the rest of the region. It was then possible to assign *p* values (Spearman rank test) for how well correlated each gene was with the rest of the region. Regions were thereby “trimmed” to the first and last gene that had a *p* value <0.05.

To calculate the FDR, gene order was randomized for the tumor dataset. A sliding window analysis of this randomized data identified seven significant TCS genes in three regions, which gives an FDR of 6.6 %.

The approach was validated against published bladder carcinoma data [19] using a sliding window algorithm of *n* = 7 (2*n* + 1 = 15 gene window). For the breast cancer z score datasets, the number of genes with significant scores (*p* < 0.05) was determined for 2*n* = 1 to 20 neighboring genes. The number of significant genes plateaued beyond *n* = 10. Therefore, a window size of *n* = 10 was used for all analysis.

To determine the number of gene neighbors significantly (*p* < 0.05) correlated with each gene with a significant TCS, a *p* value was applied to the Spearman correlation between each gene and its 20 nearest neighbors.

To determine the number of genes correlated with the genes with significant TCSs in the RER regions common to breast tumors and cell lines, the mean z score expression for each RER region in tumors/cell lines was calculated (as for the RER clustering). Lists of all genes within the RER windows were then used to ask how many of those were present on each array and showed a significant (*p* < 0.05) Spearman correlation with the mean RER expression level. Genes with significant TCSs that were in RER regions common to both breast tumors and to cell lines were based on the lists of gene symbols given in Table 1.

The mean expression level of each RER region in each sample was defined by taking the mean z score of the significant TCS genes it contains. Samples and RER regions were then clustered using the Euclidian distance and the Ward hierarchical clustering method.

### Analysis of RER region overlap with TADs

HiC TAD domain locations for hESCs, IMR90 fibroblasts and the T47D breast cancer cell line were taken from published data [43, 44]. BEDtools (v.2.17.0) was used to determine how many of these domains overlapped with the 26 RER regions defined as common to both breast tumors and cell lines [57]. To determine whether the degree of overlap exceeded that expected by chance, we randomly permutated the locations of the RER regions 1000 times (excluding known genomic gaps in the hg19 assembly) and re-assessed the degree of overlap with TADs. RER regions and permutated regions were defined as being contained within a TAD if ≥80 % of the regions span was contained within a single TAD. *P* values were defined as the percentage of times the observed overlap was seen by chance.

### Cell culture

Luminal ER+ breast cancer cell lines (MCF7, LY2, MDAMB361) and the basal ER-MDAMB231 were grown in Dulbecco's Modified Eagle's medium (DMEM), supplemented with 10 % FCS, 1 % P/S (100 units/ml penicillin, 6.5 μg/ml streptomycin). The basal cell line MDAM468 was grown in Leibovitz’s L15 medium (Gibco) instead of DMEM. HMLE normal mammary luminal epithelial cells were grown in Mammary Epithelial Growth Media (Lonza).

For hormone deprivation, FCS was stripped of all endogenous steroids. FCS (1 litre) was heat inactivated in a waterbath at 56 °C for 30 minutes before addition of 2000U/l sulfatase. The serum was incubated for 2 hours at 37 °C and then the pH adjusted to 4.2 using HCl. A charcoal mix (for 1 litre: 5 g charcoal, 25 mg dextran T70, 50 ml water) was then added and incubated overnight at 4 °C with stirring. The following day the charcoal was removed by centrifugation at 500 *g* for 30 minutes at 4 °C. The pH was then re-adjusted to 4.2 and a second charcoal mix added, incubated overnight and then removed. Centrifugation was repeated to remove any residual charcoal and the pH adjusted to 7.2 with NaOH. Stripped FCS was filter sterilized, aliquoted and stored at −20 °C.

Semi-confluent cell cultures were transferred into phenol-free DMEM (Gibco) supplemented with 5 % L-glutamine, 5 % P/S, 10 % stripped FCS and incubated for 72 hours (−E2). 17ß-estradiol (100 nM; Sigma) was then added for 24 hours (+E2).

### Fluorescence in situ hybridization

DNA hybridization probes used for FISH were fosmid probes obtained from BACPAC resources [58] and are detailed in Table 2.

**Table 2:** Fosmids probes used for FISH

Chromosomal region	Whitehead probe name	Other probe name	Start (bp)	End (bp)	Midpoint (bp)	Separation between probe pair midpoints (kb)
16p11.2 RER (sub region 1)	W12-1584N4	G248P86075G2	29,664,946	29,703,636	29,684,291	344
	W12-1754H9	G248P8656D5	30,010,045	30,046,946	30,028,496	
16p11.2 RER (sub region 2)	W12-1754H9	G248P8656D5	30,010,045	30,046,946	30,028,496	373
	W12-906G10	G248P8190D5	30,379,727	30,424,227	30,401,977	
16p11.2 RER (sub region 3)	W12-906G10	G248P8190D5	30,379,727	30,424,227	30,401,977	405
	W12-497E18	G248P8178C9	30,788,769	30,824,965	30,806,867	
16p11.2 RER (sub region 4)	W12-497E18	G248P8178C9	30,788,769	30,824,965	30,806,867	380
	W12-2222M4	G248P87014G2	31,166,763	31,207,277	31,187,020	
16p11.2 (nonRER control)	W12-3081O2	G248P89434H1	28,483,429	28,519,288	28,501,359	418
	W12-2889N9	G248P89117G5	28,899,528	28,938,582	28,919,055	
11p15.4 (nonRER control)	W12-528M6	G248P8086G3	4,961,240	4,999,789	4,980,515	493
	W12-2033J5	G248P85537E3	5,453,348	5,494,460	5,473,904	

Probe names are from the Whitehead Fosmid database [58]. Alternative probe names can be used to view fosmids on the UCSC genome browser. All genome locations (base pairs) are from the hg19 assembly of the human genome

For 2D FISH, probes were labeled with either biotin-16-dUTP or digoxigenin-11-dUTP (Roche) by nick translation then hybridized as previously described [59] but in the presence of human CotI to suppress hybridization from repetitive sequences. Labeled DNA (100–150 ng) and 12 μg of human Cot1 DNA were used per slide.

For 3D FISH on tissue sections, parrafin-embedded tissue sections were cut at 6 μm and laid on Superfrost+ slides. The slides were baked at 65 °C for 30 minutes to melt the wax, washed four times in 200 ml xylene for 10 minutes, rehydrated through an ethanol series (four 10 minute washes in each of 100 %, 95 % and 70 % ethanol) before being microwaved for a further 30 minutes in 0.1 M citrate buffer (pH 6). The slides were then allowed to cool for 20 minutes in the citrate buffer solution before being washed and stored in water. Slides were rinsed in 2× SCC before use.

Prior to hybridization, slides were washed in 2× SSC at 75 °C for 5 minutes then denatured for 3 minutes at 75 °C in 70 % formamide/2× SCC pH7.5. Slides were then placed in ice cold 100 % ethanol for 3 minutes before further dehydration in 90 % and 100 % ethanol at room temperature.

Digoxigenin-labeled probes were detected using sequential layers of fluorescein isothiocyanate (FITC)-conjugated anti-digoxygenin and FITC-conjugated anti-sheep IgG. Biotin-labeled probes were detected with sequential layers of Texas Red-conjugated avidin, biotinylated anti-avidin and Texas Red-conjugated avidin. Slides for 2D FISH were mounted in Vectashield (Vector) with 0.5 μg/ml DAPI. Slides for 3D FISH were incubated in 4× SSC/1 % Tween with 50 ng/ml DAPI for 5 minutes before mounting in Vectashield.

### Image capture

Examination of nuclei after 2D FISH was carried out using a Hamamatsu Orca AG CCD camera (Hamamatsu Photonics (UK) Ltd, Welwyn Garden City, UK) fitted to a Zeiss Axioplan II microscope with Plan-neofluar oil-immersion objectives, a 100 W Hg source and Chroma #8300 triple band pass filter set.

Examination of nuclei from tissue sections by 3D FISH was carried out using a Hamamatsu Orca AG CCD camera (Hamamatsu Photonics (UK) Ltd, Welwyn Garden City, UK), Zeiss Axioplan II fluorescence microscope with Plan-neofluar or Plan apochromat objectives, a Lumen 200 W metal halide light source (Prior Scientific Instruments, Cambridge, UK) and Chroma #89014ET single excitation and emission filters (Chroma Technology Corp., Rockingham, VT, USA) with the excitation and emission filters installed in Prior motorized filter wheels. A piezoelectrically driven objective mount (PIFOC model P-721, Physik Instrumente GmbH & Co, Karlsruhe) was used to control movement in the z dimension. Hardware control, image capture and analysis were performed using Volocity (Perkinelmer Inc, Waltham, MA, USA). Images were captured at 200 nm intervals in the z axis and were deconvolved using a calculated point spread function (PSF) with the constrained iterative algorithm of Volocity.

### Image analysis

Image capture and analysis of nuclear size, radial nuclear position and distance between the hybridization signals after 2D FISH were performed with scripts written for IPLab Spectrum (Scanalytics Copr, Fairfax, VA, USA). Scripts for analysis of 3D FISH images were carried out using scripts written for Velocity.

For 2D FISH data analysis, the mean-square inter-probe distances (d^2^) were normalized to nuclear area (r^2^) as previously described [32]. The difference between the distribution of squared inter-probe distances between datasets was assessed statistically using the Wilcox test with a cutoff of *p* < 0.05. Radial nuclear position was assessed from the proportion of hybridization signals across five concentric shells of equal area eroded from the periphery (shell 1) to the center (shell 5) of the nucleus as previously described [41, 53].

### Data availability

The following data used in this study were extracted from publically available sources. Oligonucleotide arrays (NCBI GEO, platform GPL5345) and BAC microarrays (GEO platform GPL4723) were used for global analysis of gene expression and copy number in breast tumors described in [22]. Copy number data for an additional tumor set in [23] were obtained from the Lawerence Berkley Breast Cancer lab. Gene expression profiles for these tumors are available from ArrayExpress, accession number E-TABM-158. Expression data for invasive ductal carcinoma samples and 143 breast tissue samples with normal histopathology from [28] are available from NCBI GEO under accession number GSE10780. Expression data for normal breast in [29] are under accession numbers GSE5460 and GSE7904. Expression profiles for 51 breast cancer cell lines described in [20] were obtained using Affymetrix U133A array from ArrayExpress accession number E-TABM-157. ChIP-seq data for ER from [38] are available under ArrayExpress number E-MTAB-223.

## Additional file

Additional file 1:
**Supplementary Tables S1–S4 and supplementary Figures S1–S5.** (PDF 2375 kb)

## Abbreviations

3C: chromosome conformation capture
BAC: bacterial artificial chromosome
CGH: comparative genome hybridization
ChIP: chromatin immunoprecipitation
DMEM: Dulbecco's Modified Eagle's medium
E2: 17β-estradiol
ER: estrogen receptor
ER+: estrogen receptor-positive
ER−: estrogen receptor-negative
FCS: fetal calf serum
FDR: false discovery rate
FISH: fluorescence in situ hybridization
FITC: fluorescein isothiocyanate
GEO: Gene Expression Omnibus
hESC: human embryonic stem cell
LREA: long-range epigenetic activation
LRES: long-range epigenetic silencing
PAC: P1-derived artificial chromosome
P/S: penicillin/streptomycin
RER: regional epigenetic regulation
RMA: Robust Multi-array Average
TAD: topologically associated domain
TCS: transcription correlation score
