# Supplementary material for: Estrogen-induced chromatin decondensation and nuclear re-organization linked to regional epigenetic regulation in breast cancer
Source: Genome Biol. 2015 Aug 3;16(1):145. doi: 10.1186/s13059-015-0719-9 (PMC4536608; doi:10.1186/s13059-015-0719-9)
Supplement: Additional file 1: — Supplementary Tables S1–S4 and supplementary Figures S1–S5. (PDF 2375 kb) [file 13059_2015_719_MOESM1_ESM.pdf]

**Supplementary Data**

**Table S1. RER domains generated from breast tumor data.** RERs derived from breast tumor gene expression and CGH data [22, 23]. Shown is the RER cytogenetic location, size (measured as the first to last significant TCS in the region), the number of genes with significant TCS, and the gene IDs for all the signficant TCS genes in each region.

| Cytogenetic location | Size (Mb) | No. of significant genes | Significant TCS Genes in Region |
|---|---|---|---|
| 2p24.2 - p25.1 | 2.35 | 2 | *ADAM17, PDIA6* |
| 2q14.2 - q22.1 | 2.91 | 4 | *IWS1, SAP130, UGGT1, IMP4* |
| 3p14.3-p21.31 | 4.45 | 26 | *ZNF589, CCDC51, UQCRC1, IP6K2, P4HTM, WDR6, DALRD3, IMPDH2, QARS, USP19, LAMB2, TCTA, DAG1, APEH, RNF123, IP6K1, RBM5, HYAL2, TUSC4, CYB561D2, TMEM115, MAPKAPK3, TEX264, ABHD14A, BAP1, SPCS1* |
| 4q28.2 - q31.22 | 0.29 | 2 | *SCOC, ELMOD2* |
| 6p22.1 - p22.3 | 1.39 | 3 | *C6orf62, GMNN, HIST1H4C* |
| 6p11.2 - q21 | 27 | 14 | *SLC17A5, TMEM30A, ZNF292, RARS2, MDN1, CASP8AP2, MAP3K7, KIAA0776, NDUFAF4,C6orf167, FBXL4, USP45, CCNC, ASCC3* |
| 6q23.2 - q25.3 | 14.27 | 12 | *IFNGR1, HEBP2, C6orf115, HECA, VTA1, PEX3, FUCA2, LTV1, SHPRH, PPIL4, RMND1, C6orf211* |
| 7q11.23 - q21.2 | 12.23 | 4 | *STYXL1, MDH2, DMTF1, SRI* |
| 7q33 - q36.2 | 10.94 | 11 | *SLC37A3,NDUFB2, MRPS33, SSBP1, CASP2, CUL1, EZH2, ZNF746, ABCB8, TMUB1, CHPF2* |
| 8p12 - p21.2 | 0.54 | 2 | *ZNF395, INTS9* |
| 8p11.23 - q11.23 | 3.56 | 6 | *TM2D2, GOLGA7, MYST3, AP3M2, IKBKB, C8orf40* |
| 8q21.13 - q24.3 | 42.91 | 32 | *NBN, OTUD6B, RAD54B, KIAA1429, ESRP1, INTS8, PLEKHF2, MTERFD1, PTDSS1, MTDH, HRSP12, AP003355.2, VPS13B, ANKRD46, UBR5, AZIN1, ATP6V1C1, SLC25A32, TTC35, TAF2, MRPL13, DERL1, ATAD2, WDYHV1, TRMT12, RNF139* |
| 8q24.3 | 0.99 | 13 | *TSTA3, SCRIB, PUF60, NRBP2, PARP10, GRINA, GPAA1, CYC1, SHARPIN, FBXL6, GPR172A, VPS28, CYHR1* |
| 9p13.3 – p21.3 | 1.17 | 2 | *NOL6, SIGMAR1* |
| 9q34.11 - q34.12 | 0.78 | 4 | *TRUB2, ODF2, WDR34, DOLPP1* |
| 10p12.1 – p15.1 | 5.79 | 5 | *UPF2, CDC123, HSPA14, RPP38, STAM* |
| 10q25.3 - q26.2 | 3.11 | 3 | *SEC23IP, PLEKHA1, IKZF5* |
| 11q12.2 - q13.1 | 1.709 | 5 | *C11orf48, WDR74, OTUB1, NUDT22, RPS6KA4* |
| 11q13.1 - q13.2 | 0.577 | 6 | *KAT5, FIBP, CCDC85B, SART1, SF3B2, YIF1A* |
| 12q15 - q21.33 | 8.18 | 2 | *RAB21, PPP1R12A* |

| | | | |
|---|---|---|---|
| 13q14.11 - q14.2 | 1.32 | 2 | *ESD, MED4* |
| 13q23.3 - q32.11 | 4.81 | 3 | *COMMD6, UCHL3, SPRY2* |
| 13q33.1 - q34 | 0.42 | 2 | *ANKRD10, ARHGEF7* |
| 14q11.2 - q12 | 2.83 | 7 | *CHD8, RBM23, PCK2, DCAF11, RNF31, IPO4, CHMP4A* |
| 14q23.3 - q32.11 | 8.36 | 8 | *SLC39A9, COX16, NUMB, ZNF410, COQ6, ENTPD5, GSTZ1, SNW1* |
| 16p13.3 | 1.86 | 23 | *TMEM8A, NME4, RAB11FIP3, PIGQ, RAB40C, LA16c-398G5.2, WDR90, RHOT2, WDR24, METRN, FAM173A, CCDC78, NARFL, IFT140, NME3, MRPS34, HAGH, NDUFB10, GFER, NTHL1, TRAF7, MLST8, E4F1* |
| 16p13.13 - p13.3 | 0.45 | 4 | *DNAJA3, ANKS3, ROGDI, UBN1* |
| 16p12.1 - p12.3 | 0.12 | 3 | *EARS2, NDUFAB1, PALB2* |
| 16p11.2 | 0.53 | 6 | *TBC1D10B, ZNF48, PRR14, FBRS, PHKG2, BCL7C* |
| 16q12.2 - q24.1 | 23.58 | 22 | *COQ9, C16orf57, C16orf80, CMTM1, DYNC1LI2, CES2, TMEM208, ACD, CENPT, SLC7A6OS, CIRH1A, WWP2, AARS, DDX19A, COG4, SF3B3, FTSJD1, DHX38, PSMD7, TMEM170A, ADAT1, CENPN* |
| 16q24.1 - q24.3 | 0.51 | 6 | *CYBA, MVD, CTU2, FAM38A, CDT1, ACSF3* |
| 17p13.2 - p13.3 | 0.16 | 2 | *MYBBP1A, PELP1* |
| 17p12 - q11.2 | 10.47 | 17 | *COPS3, MED9, SMCR8, AKAP10, IFT20, POLDIP2, UNC119, PIGS, SPAG5, KIAA0100, SDF2, SUPT6H, TLCD1, ERAL1, FLOT2, DHRS13, NUFIP2* |
| 17q12 - q21.2 | 2.44 | 17 | *TADA2A, MRPL45, MLLT6, PIP5K2B, CWC25, FBXL20, MED1, CRKRS, STARD3, PGAP3, ERBB2, C17orf37, GRB7, GSDMB, ORMDL3, PSMD3, MED24* |
| 17q21.2 - q21.31 | 0.79 | 7 | *GHDC, COASY, FAM134C, VPS25, CCDC56, PSME3, AARSD1* |
| 17q21.32 - q24.1 | 13.73 | 33 | *PDK2, PPP1R9B, MRPL27, LRRC59, RSAD1, TOB1, MSI2, MRPS23, CUEDC1, SFRS1, DYNLL2, RNF43, MTMR4, RAD51C, TRIM37, C17orf71, DHX40, CLTC, PTRH2, TMEM49, TUBD1, HEATR6, USP32, APPBP2, BCAS3, BRIP1, MED13, DCAF7, STRADA, CCDC47, DDX42, FTSJ3, PSMC5* |
| 17q25.1 - q25.3 | 2.72 | 24 | *TMEM104, ICT1, KCTD2, ARMC7, NT5C, HN1, NUP85, MIF4GD, SLC25A19, GRB2, KIAA0195, SAP30BP, GALK1, WBP2, TRIM47, TRIM65, FBF1, C17orf106, EVPL, SRP68, PRPSAP1, UBE2O, RHBDF2, SEPT9* |
| 17q25.3 | 1 | 13 | *AZI1, FSCN2, C17orf70, NPLOC4, HGS, MRPL12, ASPSCR1, STRA13, LRRC45, DCXR, RFNG, GPS1, CCDC57* |
| 18p11.22 - q11.2 | 1.06 | 5 | *PSMG2, PTPN2, CEP192, C18orf19, RNMT* |
| 18q12.3 - q21.32 | 7.14 | 3 | *IER3IP1, SMAD4, POLI* |
| 20q13.2 - q13.33 | 3.82 | 5 | *RAB22A, VAPB, GNAS, TH1L, LSM14B* |
| 22q11.23 - q12.1 | 1.18 | 5 | *C22orf13, SNRPD3, C22orf36, KIAA1671, ADRBK2* |
| 22q12.2 - q12.3 | 3.03 | 3 | *C22orf28, FBXO7, MCM5* |
| 22q13.1 - q13.2 | 0.31 | 2 | *ZC3H7B, PPPDE2* |
| 22q13.31 - q13.33 | 0.32 | 2 | *CRELD2, TRABD* |

**Table S2: RER domains generated from breast cancer cell lines.** RER analysis derived from data in breast cancer cell lines [20]. Shown is the cytogenetic location of each RER, the RER size measured from the first to last significant TCS in the region, the number of significant genes and the gene IDs for all the significant TCS genes in the region.

| Cytogenetic position | Size (Mb) | No. of significant genes | Significant TCS Genes in Region |
|---|---|---|---|
| 1p36.13 | 0.53 | 2 | *AKR7A3, RNF186* |
| 1p34.3 | 0.33 | 3 | *CDCA8,YRDC,UTP11L* |
| 1p34.1 - p34.2 | 0.63 | 3 | *ELOVL1,ATP6V0B,B4GALT2* |
| 1q21.3 - q23.3 | 7.55 | 42 | *SPRR1B,S100A14,SLC39A1,JTB,C1orf43,UBAP2L,HAX1,UBE2Q 1,ADAR,PMVK,ADAM15,EFNA3,EFNA1,RAG1AP1,DPM3,MUC1, SCAMP3,CLK2,RUSC1,YY1AP1,DAP3,ROBLD3,RAB25,MEF2D, GPATCH4,BCAN,CD1C,CD1B,CD1E,CADM3,CTA34P22.2,DARC ,OR10J1,APCS,CRP,SLAMF8,CCDC19,KCNJ10,KCNJ9,ATP1A2, CASQ1,CD84* |
| 1q42.2 | 0.37 | 2 | *TTC13, GNPAT* |
| 1q43 | 0.39 | 2 | *FH,EXO1* |
| 2q32.3 - q33.1 | 9.66 | 4 | *MYO1B, HSPE1, C2orf47, NIF3L1* |
| 3p22.2 | 0.33 | 2 | *VILL, SLC22A14* |
| 3p21.31 – p22.1 | 3.46 | 9 | *ANO10, ZDHHC3, SLC6A20, FYCO1, CCR1, CCR2, CCR5, CCRL2, PRSS50* |
| 4p16.3 | 2.05 | 11 | *TACC3, WHSC1, WHSC2, HAUS3, RNF4, TNIP2, SH3BP2, ADD1, TETRAN, HTT, ADRA2C* |
| 4q13.2 - q13.3 | 5.11 | 11 | *UGT2A3, SULT1B1, CSN1S1,CSN2, STATH, CSN3, SMR3B, PROL1, IL8, CXCL5, CXCL3* |
| 4q22.1 | 0.40 | 2 | *MEPE, ABCG2* |
| 4q26 - q27 | 2.09 | 3 | *MYOZ2,PDE5A,TNIP3* |
| 5p13.2 | 2.47 | 4 | *RAD1,BRIX1,SKP2,NUP155* |
| 6p22.1 - p22.2 | 3.61 | 34 | *HIST1H4B,HIST1H3B,HIST1H2AB,HIST1H2BB,HIST1H3C, HIST1H1C,HIST1H2BC,HIST1H2AC,HIST1H1E,HIST1H2BD ,HIST1H2BE,HIST1H4D,HIST1H3D,HIST1H2BF,HIST1H4E, HIST1H2BG,HIST1H2AE,HIST1H1D,HIST1H3F,HIST1H2BH, HIST1H2APS4,HIST1H2BI,HIST1H4H,HIST1H2BM,HIST1H2AM ,HIST1H2BO,ZNF435,GPX5,OR2W1,OR2J3,OR2N1P,OR12D2, OR10C1,MOG* |
| 6p21.33 | 0.07 | 4 | *LST1,NCR3,BAT2,APOM* |
| 6p21.32 - 6p21.33 | 0.96 | | *TNXB, CREBL1, PPT2, GPSM3, NOTCH4, C6orf10, HLA-DRB6, HLA-DQB2, HLA-DMA* |
| 6q23.2 | 0.15 | 3 | *TAAR5,TAAR3,VNN3* |
| 6q25.2 | 1.35 | 3 | *RP3-468K3.1, RP3-527B10.1,OPRM1* |
| 6q26 - q27 | 6.43 | 7 | *PARK2,PDE10A,T,CCR6,GPR31,TCP10,C6orf123* |
| 7p22.1 - p22.3 | 3.97 | 6 | *MAD1L1,FTSJ2,CHST12,FOXK1,ACTB,RNF216* |
| 7q22.1 | 0.15 | 2 | *EPO,SLC12A9* |
| 8p21.3 | 0.90 | 6 | *BMP1,POLR3D,PIWIL2,SLC39A14,PPP3CC,SORBS3,BIN3, RHOBTB2,TNFRSF10B* |

| | | | |
|---|---|---|---|
| 8p11.21 - q11.23 | 13.05 | 6 | *AP3M2,POLB,VDAC3,SLC20A2,MCM4,MRPL15* |
| 8q13.3 - q21.3 | 15.86 | 6 | *KCNB2,STAU2,FAM164A,STMN2,FAM82B,MMP16* |
| 8q22.2 - q22.3 | 5.29 | 11 | *RPL30,HRSP12,NIPAL2,VPS13B,COX6C,SPAG1,RNF19A, ANKRD46,UBR5,AZIN1,FZD6* |
| 9p13.3 | 0.72 | 6 | *KIAA1045,DNAJB5,RUSC2,CD72,SIT1,CA9* |
| 9q34.3 | 2.10 | 5 | *OLFM1,C9orf116,SNAPC4,ABCA2,GRIN1* |
| 10p13 | 1.98 | 3 | *HSPA14,NMT2,RSU1* |
| 10q22.1 - q22.2 | 2.00 | 4 | *PSAP,CBARA1,SEC24C,NDST2* |
| 10q23.33 | 0.39 | 2 | *CYP2C18, CYP2C8* |
| 10q26.11 - q26.13 | 2.78 | 5 | *BAG3,C10orf119,SEC23IP,BRWD2,PLEKHA1* |
| 11q12.1 - q12.2 | 0.93 | 6 | *MS4A2,MS4A5,MS4A12,GPR44,TMEM109,CD6* |
| 11q13.1 | 0.19 | 2 | *COX8A,MACROD1* |
| 11q13.2 | 0.16 | 2 | *SPTBN2,C11orf80* |
| 11q23.3 - q24.3 | 11.11 | 14 | *FXYD6,CD3G,MLL,H2AFX,PDZD3,THY1,TECTA, SCN3B,ACRV1,DDX25,CDON,KCNJ1,KCNJ5,TP53AIP1* |
| 12p13.32 | 0.16 | 2 | *AKAP3,GALNT8* |
| 12p13.31 | 0.95 | 3 | *C3AR1,AICDA,KLRG1* |
| 12p13.2 | 1.46 | 2 | *TAS2R9,LRP6* |
| 12q13.11 - q13.12 | 0.72 | 3 | *ASB8,CACNB3,RND1* |
| 12q13.2 | 1.29 | 4 | *PDE1B,BLOC1S1,RDH5,MMP19* |
| 12q21.31 - q22 | 7.06 | 4 | *NTS,MGAT4C,DCN,EEA1* |
| 14q32.13 | 0.23 | 3 | *SERPINA2,SERPINA4,SERPINA5* |
| 16p13.3 | 1.95 | 23 | *AXIN1,TMEM8A,NME4,RAB11FIP3,PIGQ,RAB40C ,RHOT2,FAM173A,NARFL,GNG13,LMF1,CACNA1H, TPSG1,TPSD1,UBE2I,BAIAP3,NME3,MRPS34,NUBP2, GFER,NTHL1,PGP,E4F1* |
| 16p13.3 | 1.07 | 5 | *OR1F1,OR2C1,NAT15,ADCY9,TFAP4* |
| 16p12.3 | 0.62 | 2 | *C16orf88,GP2* |
| 16p11.2 | 1.22 | 25 | *SEZ6L2,TAOK2,HIRIP3,DOC2A,ALDOA,PPP4C,TBX6, MAPK3,CD2BP2,TBC1D10B,ZNF771,ZNF768,ZNF747, ZNF764,ZNF688,ZNF785,PRR14,FBRS,SRCAP,PHKG2, RNF40,ZNF629,CTF1,SETD1A,VKORC1* |
| 16q22.1 - q22.2 | 3.89 | 11 | *CDH16,NOL3,E2F4,ATP6V0D1,THAP11,PSKH1,DDX28 ,DUS2L,PRMT7,COG4,VAC14* |
| 17p11.2 | 0.40 | 3 | *TOM1L2,LRRC48,LLGL1* |
| 17q11.2 | 0.16 | 4 | *UNC119,KIAA0100,SDF2,SUPT6H* |
| 17q21.2 | 0.36 | 6 | *KRTAP1-3,KRTAP1-1,KRTAP2-4,KRTAP4-9,KRT34 ,KRT31* |
| 17q21.31 | 0.66 | 2 | *RUNDC3A,C1QL1* |
| 17q25.1 | 0.36 | 4 | *KCTD2,GGA3,MRPS7,GRB2* |
| 17q25.3 | 0.32 | 4 | *STRA13,RFNG,CSNK1D,SECTM1* |
| 19p13.3 | 0.66 | 4 | *PIAS4,ZBTB7A,SH3GL1,C19orf10* |
| 19p13.2 | 0.03 | 2 | *MAP2K7,SNAPC2* |
| 19p13.12 - p13.2 | 5.06 | 23 | *CDC37,TMED1,AC024575.1,EPOR,ELAVL3,CNN1, ACP5,C19orf56,TNPO2,ASNA1,BEST2,PRDX2,RNASEH2A ,MAST1,DNASE2,GCDH,GADD45GIP1,PRKACA,ASF1B ,OR7C2,SLC1A6,EPHX3,WIZ* |
| 19p13.11 | 1.33 | 8 | *SLC5A5,PIK3R2,PGPEP1,ELL,FKBP8,DDX49,TMEM161A ,RFXANK* |
| 19q13.12 | 0.55 | 6 | *CD22,GAPDHS,ZBTB32,ARHGAP33,NPHS1,APLP1* |

| | | | |
|---|---|---|---|
| 19q13.2 | 0.72 | 7 | *CD79A,GRIK5,ZNF574,POU2F2,GSK3A,CIC,CEACAM8* |
| 19q13.33 - q13.42 | 4.53 | 37 | *SHANK1,KLK1,KLK3,KLK2,KLK6,KLK11,KLK12,KLK13, KLK14,SIGLEC9,SIGLEC7,CD33,NKG7,LIM2,SIGLEC6, ZNF175,SIGLEC5,HAS1,FPR1,FPR2,FPR3,ZNF528,LILRB3, LILRB5,LILRA3,LILRA5,LAIR1,KIR3DX1,LILRB1,LILRB4, AC006293.3,KIR2DL4,KIR3DL1,FCAR,NCR1,GP6,SYT5* |
| 19q13.43 | 0.46 | 5 | *ZNF550,ZNF134,ZNF211,ZNF586,ZNF606* |
| 20p13 | 0.01 | 2 | *OXT, AVP* |
| 20p11.11 - p11.23 | 7.38 | 14 | *RBBP9,INSM1,FOXA2,CYB5P4,THBD,CD93,CST8,CST3, CST4,CST5,TMEM90B,CST7,C20orf3,FAM182B* |
| 20q13.12 | 0.48 | 4 | *ZSWIM1,MMP9,CDH22,SLC35C2* |
| 20q13.33 | 0.82 | 3 | *DIDO1,ARFGAP1,RTEL1* |
| 21q22.3 | 0.67 | 2 | *RRP1, LRRC3* |
| 22q11.21 - q12.1 | 6.91 | 13 | *TRMT2A,P2RX6,TOP3B,PPIL2,IGLV1-40,ZNF280B,ZNF280A, ZDHHC8P,VPREB3,MMP11,UPB1,SEZ6L,CRYBB1* |
| 22q12.2 | 0.17 | 2 | *INPP5J, PIK3IP1* |
| 22q12.3 - q13.1 | 0.75 | 4 | *TMPRSS6,SSTR3,MFNG,GCAT* |
| 22q13.1 | 1.55 | 6 | *CBX6,APOBEC3A,PDGFB,MGAT3,CACNA1I,SGSM3* |

**Table S3**: Wilcox test p-values for cell line analysis of normalised interprobe distances for locus 2 in the RER on chromosome 16p11.2. Relates to Figure 5A.

| Cell lines tested | | Wilcox test p-value |
|---|---|---|
| HMLE | MCF7 | 0.001 |
| HMLE | LY2 | 4.31E-09 |
| HMLE | MDAMB361 | 0.03 |
| HMLE | MDAMB231 | 0.20 |
| HMLE | MDAMB468 | 0.07 |
| MCF7 | LY2 | 0.02 |
| MCF7 | MDAMB361 | 0.13 |
| MCF7 | MDAMB231 | 0.03 |
| MCF7 | MDAMB468 | 0.04 |
| LY2 | MDAMB361 | 6.49E-06 |
| LY2 | MDAMB231 | 4.80E-07 |
| LY2 | MDAMB468 | 7.26E-07 |
| MDAMB361 | MDAMB231 | 0.36 |
| MDAMB361 | MDAMB468 | 0.72 |
| MDAMB231 | MDAMB468 | 0.47 |

**Table S4:** Summary of comparison between LRES domains in bladder carcinoma with RER domains in breast cancer cell lines and tumors

| Bladder TCS region (identification number) | Cytoband | Significant TCS genes in bladder carcinoma (after recalculation) | Size (bp) in bladder carcinoma | Breast Tumor RER | Breast Cell line RER (identification number) |
|---|---|---|---|---|---|
| 4-2 | 4q13.3 | *IL8, CXCL6, CXCL1, CXCL2, CXCL5, CXCL3* | 296 | - | √ |
| 6-7 | 6q23.3–6q24.1 | *MAP7, MAP3K5, PEX7, IFNGR1, HEBP2, C6orf80, CITED2* | 3029 | √ | - |
| 14-1 | 14q11.2 | *LRP10, ACIN1, PABPN1, EFS, AP1G2, DHRS2,PCK2, WDR23, PSME1* | 1,260 | √ | - |
| 19-2 | 19p13.13 | *PRDX2, FARSLA* | 125 | - | √ |
| 19-3 | 19p13.12–19p13.11 | *WIZ, CYP4F3, CYP4F12, CYP4F11, CHERP, SIN3B, MYO9B,FLJ22709, NR2F6, FCHO1, B3GNT3, INSL3, SLC5A5, PIK3R2* | 2,732 | - | √ |
| 6-5 | 6q16.1 | *KIAA0776, C6orf111, ASCC3, PREP* | 8,755 | √ | - |
| 1-4 | 1p34.1 | *ATP6V0B, PRNPIP, (RPS15A), (UROD), (PRDX1), (AKR1A1)* | 2,108 | - | √ |
| 17-8 | 17q21.33–17q22 | *WDR50, COX11* | 3,701 | √ | - |
| 3-2 | 3p22.3 | *PLCD1, ACAA1, MYD88* | 277 | - | √ |
| 3-5 | 3p21.31 | *IMPDH2, GPX1, RHOA, AMT, UBE1L, MST1R* | 1,288 | √ | - |

## Supplementary Figures

**Figure S1. Transcription Correlation Score (TCS) maps in breast tumors**

Transcription Correlation Score (TCS) maps for all chromosomes using data from breast tumors [22]. The horizontal dotted line indicates the significance threshold.

**Figure S2. Characteristics of genes in breast cancer RERs**

A) Box plots showing the distribution of gene densities (gene/Mb) for all genes analysed and for genes with significant TCSs in breast cancer. Genes with significant TCSs are in regions of the genome that are significantly more gene dense than expected ($p=2.2e^{-16}$).

B) Analysis of Gene Ontology (GO) associated with the significant TCS genes.

**Figure S3. Transcription Correlation Score (TCS) maps in breast tumors**

Transcription Correlation Score (TCS) maps for all chromosomes using data from breast cancer cell lines [20]. The horizontal dotted line indicates the significance threshold.

**Figure S4. Absence of chromatin compaction differences at a control locus**

Example FISH images using control probe pairs (red and green) from a non-RER region in ER+ve cell lines MCF7 and LY2 (upper panels), and ER-ve cell lines MDAMB231 and MDAMB468 (lower panels). DNA is stained with DAPI (blue). Scale bar = 5μm. The boxplots to the right show the distribution of normalised FISH interprobe distances ($d^2/r^2$) in the four cell lines. n = 45-60 nuclei.

**Figure S5. Absence of E2-induced chromatin de-compaction at a control locus**

Box plots comparing the distribution of normalised FISH interprobe distances ($d^2/r^2$) measured across a control region in ER+ve MCF7 breast cancer cell lines. Data are shown for cells grown in normal serum, in media stripped of hormone for 3 days (-E2), and after addition of 100nm estrogen for 24 hours (+E2). n = 60 cells for each sample.

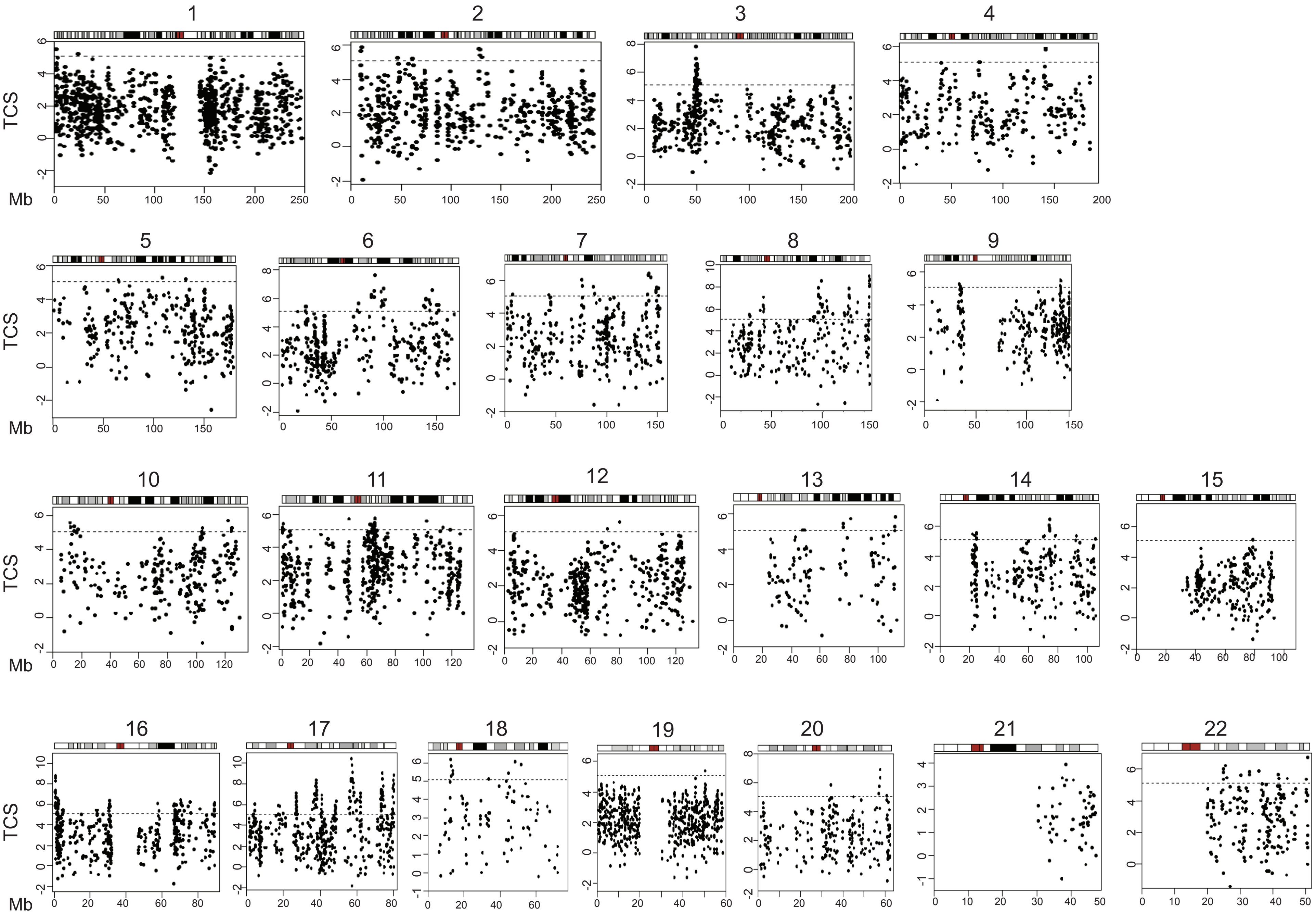


Figure S1

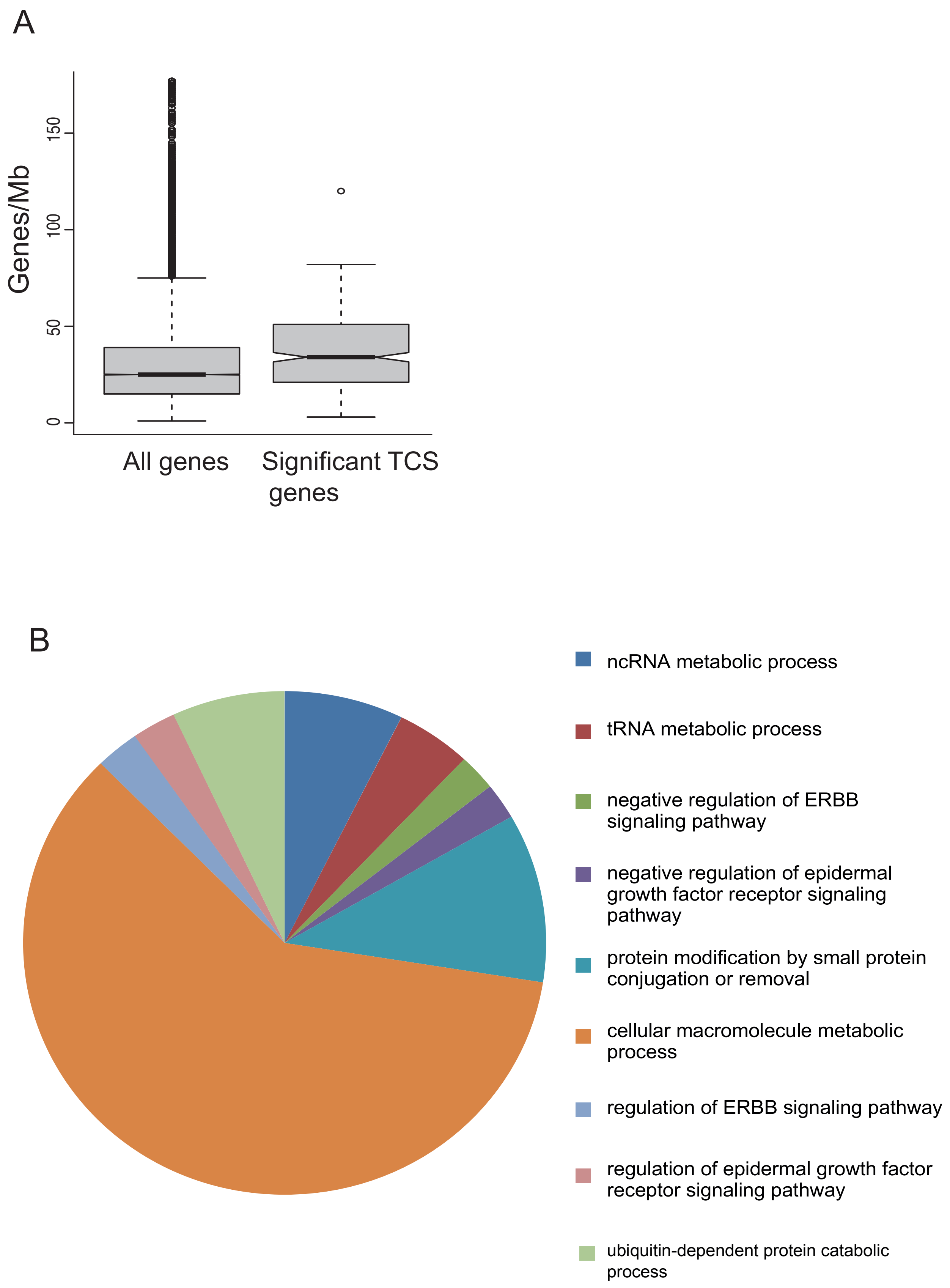
A
Genes/Mb
0
50
100
150
All genes
Significant TCS genes
B
ncRNA metabolic process
tRNA metabolic process
negative regulation of ERBB signaling pathway
negative regulation of epidermal growth factor receptor signaling pathway
protein modification by small protein conjugation or removal
cellular macromolecule metabolic process
regulation of ERBB signaling pathway
regulation of epidermal growth factor receptor signaling pathway
ubiquitin-dependent protein catabolic process

Fig. S2

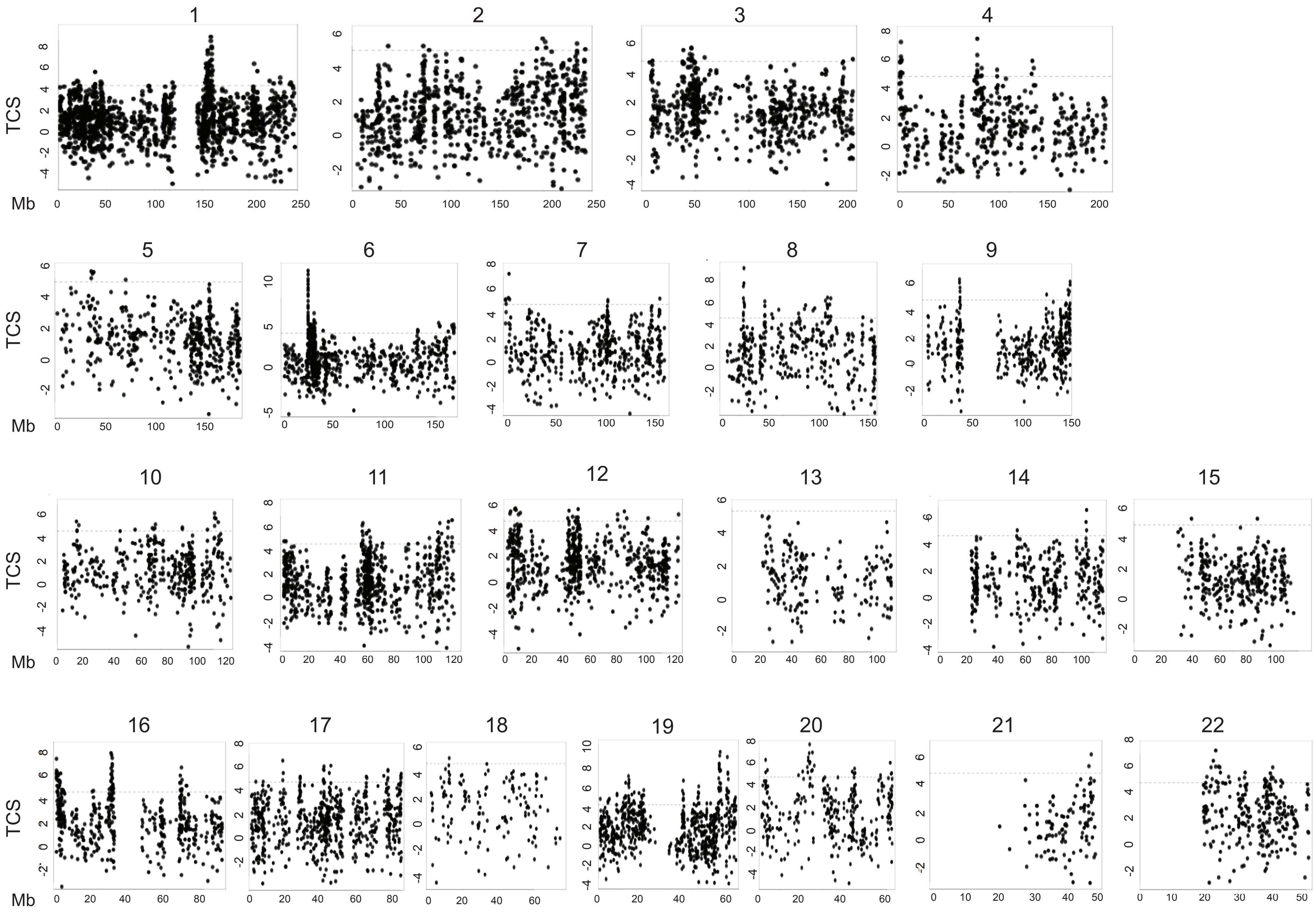


Figure S3

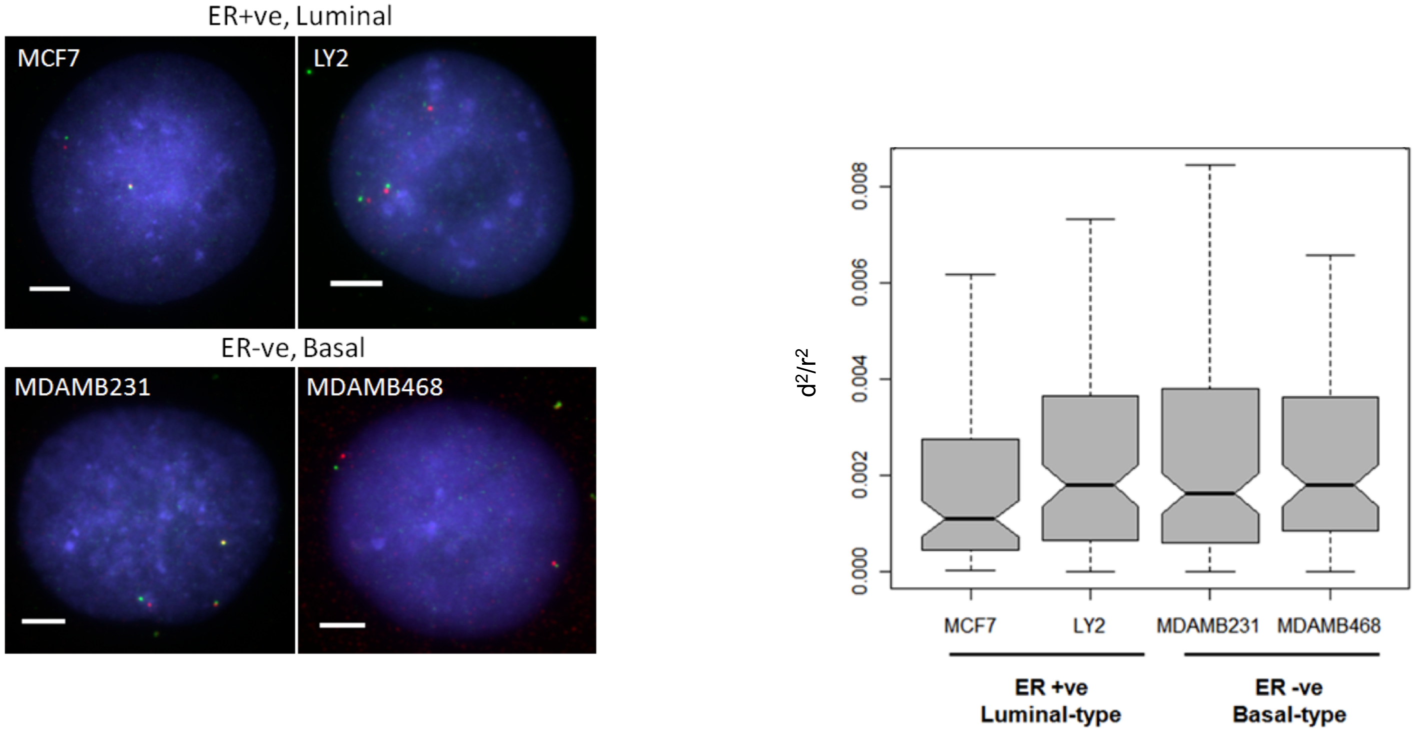
ER+ve, Luminal
MCF7
LY2
ER-ve, Basal
MDAMB231
MDAMB468
d2/r2
0.000
0.002
0.004
0.006
0.008
MCF7
LY2
MDAMB231
MDAMB468
ER +ve
Luminal-type
ER -ve
Basal-type

Fig S4

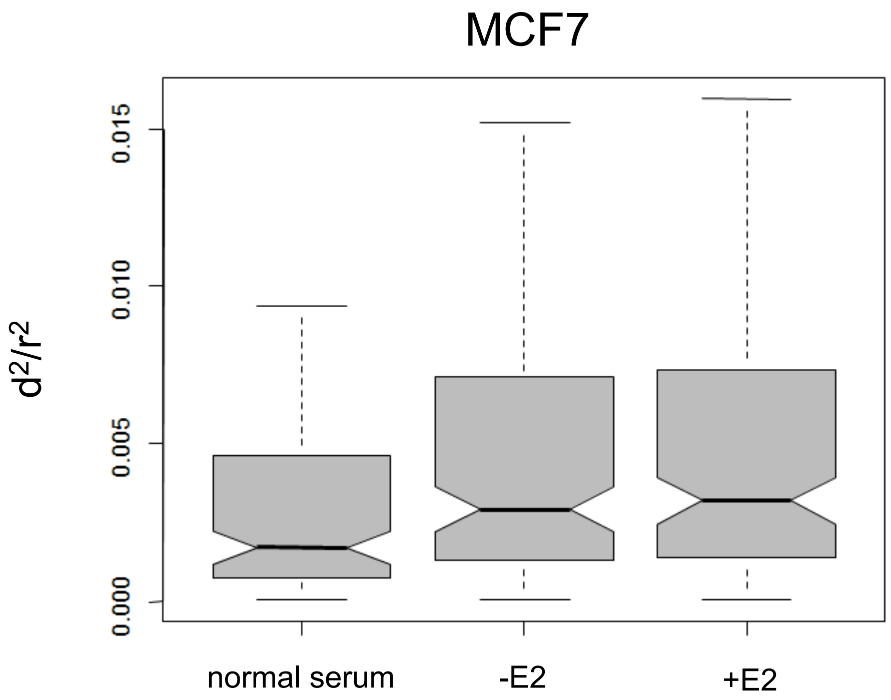


Fig S5
